# The use of chiral lithium amides in the desymmetrisation of *N*-trialkylsilyl dimethyl sulfoximines

**DOI:** 10.1186/1860-5397-3-33

**Published:** 2007-10-16

**Authors:** Matthew J McGrath, Carsten Bolm

**Affiliations:** 1 Institut für Organische Chemie, Landoltweg 1, RWTH Aachen, Aachen 52074, Germany

## Abstract

**Background:**

Chiral base desymmetrisation of dimethyl sulfoximines could provide a general route to chiral, enantioenriched dialkyl sulfoximines with potential for use in asymmetric catalysis.

**Results:**

Asymmetric deprotonation of *N*-trialkylsilyl dimethyl sulfoximines with either enantiomer of lithium *N*,*N*-bis(1-phenylethyl)amide in the presence of lithium chloride affords enantioenriched sulfoximines on electrophilic trapping. Ketones, ketimines, trialkylsilyl chlorides and activated alkyl halides may be used as electrophiles in the reaction. Furthermore, a modified Horner-Emmons methodology was investigated.

**Conclusion:**

Simple chiral lithium amides afford products with enantiomeric excesses of up to 70%, illustrating that chiral base desymmetrisation of dimethyl sulfoximines is possible.

## Introduction

The preparation of enantioenriched sulfoximines is an important goal in synthesis as these *S-*chiral compounds make interesting ligands for asymmetric catalysis and have been used in the construction of pseudopeptides. [1–4] Enantiomerically enriched aryl-alkylsulfoximines are generally prepared by resolution with camphorsulfonic acid but this method is not applicable to dialkyl sulfoximines. [5–7]

Imination of enantioenriched sulfoxides and derivatisation of sulfoximines with enantiomerically pure amino acid derivatives also provide enantioenriched *S*-chiral sulfoximines but both methods lack generality due to the limited availability of the appropriate chiral sulfoxides and the limited scope for cleavage of the chiral auxiliary in the latter process. [6–15]

**Scheme 1:**
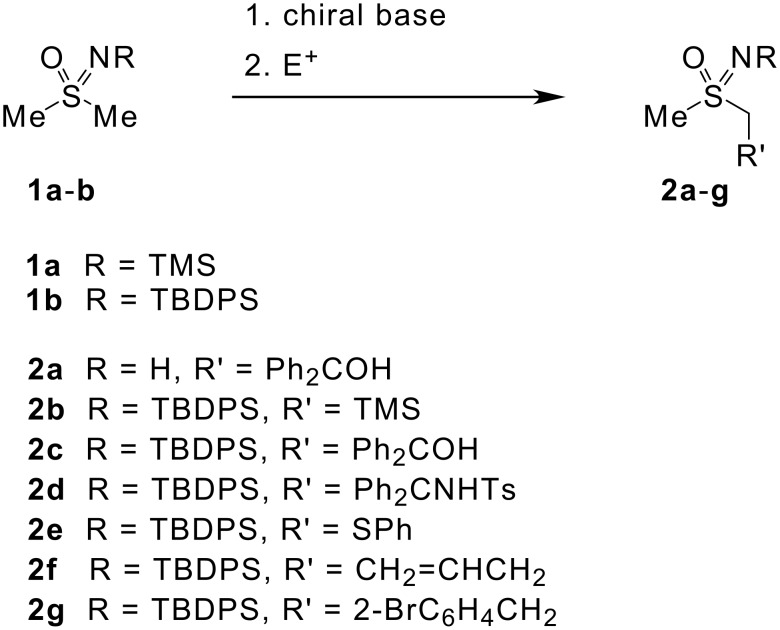
Desymmetrisation of *N*-trialkylsilyldimethylsulfoximines using chiral bases

Selective substitution of one of the enantiotopic methyl groups in a dimethylsulfoximine derivative **1,** by treatment with a chiral base followed by electrophilic trapping of the resultant anion, would provide an alternative access to enantioenriched sulfoximines of type **2**, which are not readily accessible by other means (Scheme 1). [16–19]

## Results and Discussion

*N*-Trialkylsilyl protected sulfoximines were chosen as candidates for chiral base desymmetrisation as simple variation of the silyl protecting group allows for tuning of the steric bulk at the sulfoximine nitrogen and because of the potential for subsequently unmasking the sulfoximine NH under mild conditions.

**Scheme 2:**
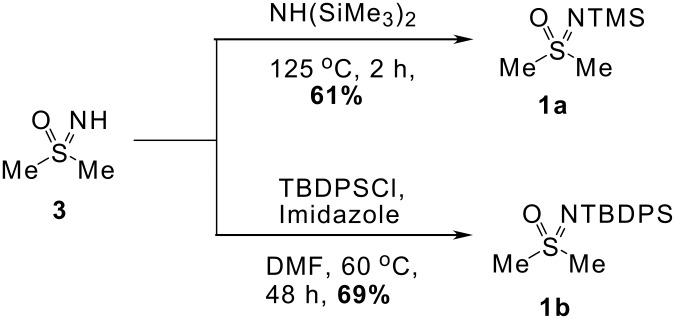
Preparation of *N*-trialkylsilyl dimethyl sulfoximine derivatives

*N*-Protected sulfoximines were prepared from dimethyl sulfoximine in a straightforward manner (Scheme 2). Treatment of dimethyl sulfoximine (**3**) with hexamethyldisilazane or with *tert*-butyldiphenylsilyl chloride and imidazole in DMF gave the silylated sulfoximines **1a** and **1b** in 61% and 69% yield, respectively, thus furnishing substrates for deprotonation reactions.

**Figure 1:**
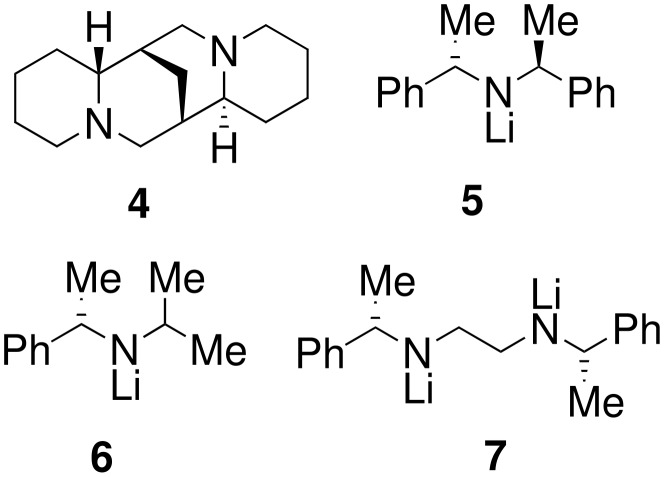
The diamine (-)-sparteine and three chiral lithium amide bases.

Initial attempts at enantioselective deprotonation of TMS protected **1a** using a complex of (-)-sparteine **4** (Figure 1) and *n*-BuLi as the chiral base and trapping the resulting anion with benzophenone gave the desilylated *β*-hydroxysulfoximine **2a** with low enantiomeric excesses (Table 1, Entries 1 and 2).

**Table 1:** Enantioselective Deprotonation of Trialkylsilyl-protected Sulfoximines 1: Optimisation of Reaction Conditions

Entry	SM	Electrophile	Base	Solvent	T (°C)	Product	Yield (%)	ee (%)^a^

1	**1a**	**Ph**_2_**CO**	**4**•*n*-BuLi	Et_2_O	-78	**2a**	41	8
2	**1a**	**Ph**_2_**CO**	**4**•*n*-BuLi	Toluene	-78	**2a**	27	2
3	**1b**	**TMSCl**	**4**•*s*-BuLi	Et_2_O	-78	**2b**	41	7
4	**1a**	**Ph**_2_**CO**	**5**•LiCl	THF	-78	**2a**	70^b^	-42^c^
5	**1b**	**TMSCl**	**5**•LiCl	THF	-78	**2b**	72	36
6	**1b**	**TMSCl**	**5**•LiCl	THF	-94	**2b**	27	-54^c^
7	**1b**	**TMSCl**	**5**•LiCl	THF	-105	**2b**	58	61
8	**1b**	**Ph**_2_**CO**	**6**•LiCl	THF	-105	**2c**	41	38
9	**1b**	**TMSCl**	**7**•2LiCl	THF	-105	**2b**	53	0

^a^ Determined by HPLC using a chiral column. ^b^ Determined by NMR integration, all other yields refer to the amount of isolated product. ^c^ Denotes use of (*R*,*R*)-**5 (*S,S*)-5** in all other cases.

Similarly a combination of (-)-**4**/*s*-BuLi was unselective in the desymmetrisation of silyl derivative **1b**. Thus using TMSCl as electrophile, adduct **2b** was obtained in 41% yield with 7% ee (Table 1, Entry 3). Deprotonation with lithium amide (*R,R*)-**5** in the presence of lithium chloride (conveniently generated *in situ* by deprotonation of the amine hydrochloride with *n*-BuLi [20–21] gave an inseparable mixture of **2a** and *bis*(1-phenylethyl)amine, encouragingly however, HPLC examination of the mixture using a chiral column indicated that adduct **2a** was formed in 42% ee (Table 1, entry 4). To overcome the separation problem, *N-tert*-butyldiphenylsilyl dimethylsulfoximine **1b** was used in place of trimethylsilyl adduct **1a** and the electrophilic trap was changed to TMSCl. Under these modified conditions, the chiral amine could be removed from the product mixture by extraction with aqueous phosphoric acid and a 72% yield of the TBDPS adduct **2b** was obtained in a promising 36% ee (Table 1, entry 5).

Next, attention was focused on optimising the reaction temperature and the chiral lithium amide structure. Lowering the deprotonation temperature to -94°C (MeOH-liquid nitrogen bath) gave silyl adduct **2b** in 54% ee (Table 1, Entry 6), a further decrease in temperature to -105°C gave the desired adduct in 61% ee and 58% yield (Table 1, Entry 7). Use of lithium amide **6** with benzophenone as electrophile led to adduct **2c** of only 38% ee (Table 1, Entry 8) and reactions with lithium diamide **7** gave silyl derivative **2b** in good yield but with no detecenantiomeric excess (Table 1, Entry 9).

Various electrophiles were tested in the desymmetrisation reaction using lithium amide **5**. Benzophenone proved to be superior to TMSCl (Table 2, Entry 1), affording **2c** in high yield and improved ee (86% yield, 70% ee, Table 2, Entry 2). When *N*-(diphenylmethylene)-toluenesulfonamide was used as electrophile the sulfoximine adduct **2d** was obtained in low yield and ee (Table 2, Entry 3). In contrast, diphenyl disulfide gave sulfide **2e** in accepee but in low yield (Table 2, Entry 4) presumably due to facile deprotonation of the product under the reaction conditions. A moderate yield was also obtained on allylation with allyl iodide (Table 2, Entry 5) but in this case the enantiomers could not be resolved by HPLC using a chiral column. A benzylic halide was also reactive thus 2-bromobenzyl bromide gave adduct **2g** in 70% yield, 57% ee (Table 2, Entry 6).

**Table 2:** Scope of Dimethylsulfoximine Desymmetrisation Reaction Using Lithium Amide 5^a^

Entry	Electrophile	Product	Yield (%)	ee (%)^b^

1	TMSCl	**2b**	58	61
2	Ph_2_CO	**2c**	86	70
3	Ph_2_C = NTs	**2d**	30	28
4	PhSSPh	**2e**	6	62
5	CH_2_ = CHCH_2_I	**2f**	34	-^c^
6	2-BrC_6_H_4_CH_2_Br	**2g**	70	57

^a^ Reactions were performed using sulfoximine **1b** and an excess of lithium amide **5** at -105°C in THF see Supporting Information File 1 for experimental details ^b^ Determined by HPLC using a chiral column. ^c^ Not determined

**Scheme 3:**
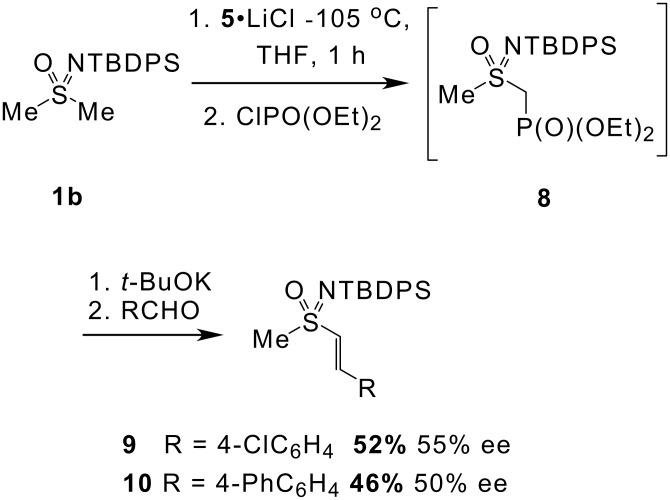
Preparation of vinyl sulfoximines

Vinyl sulfoximines could be accessed by trapping the lithiated sulfoximine with diethyl chlorophosphate (presumably affording **8**) followed by treatment with *t*-BuOK and an aldehyde in an *in situ* Horner Emmons process (Scheme 3). [22] Use of 4-chlorobenzaldehyde or 4-phenylbenzaldehyde gave *E-*alkenes **9** and **10** in moderate yields and enantiomeric excesses.

**Figure 2:**
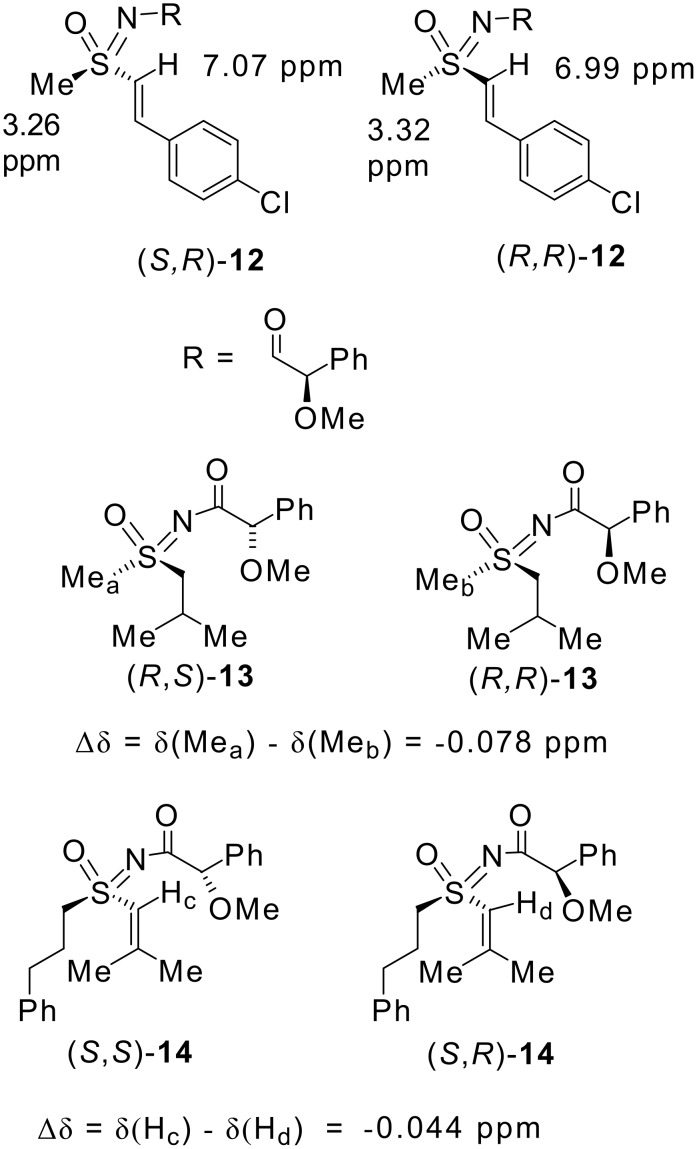
Selected NMR Characteristics of sulfoximine amides **12, 13** and **14**.

**Scheme 4:**
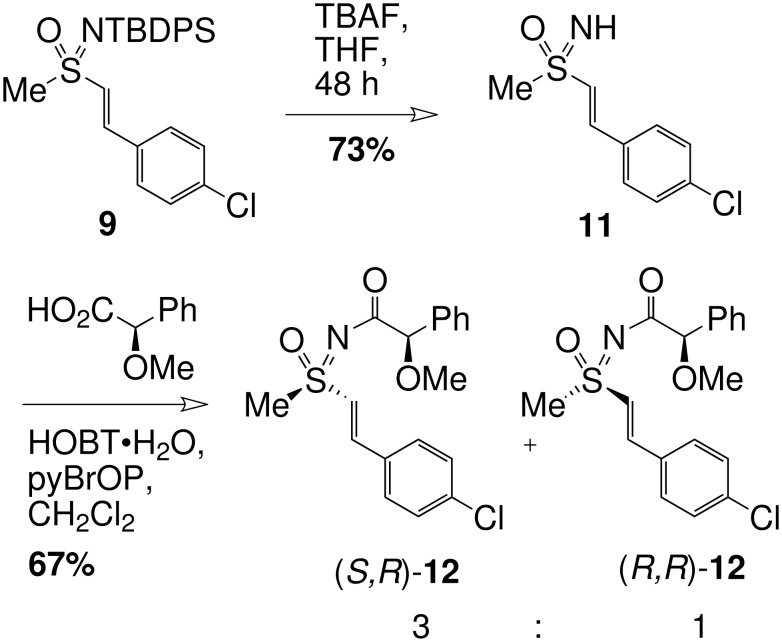
Preparation of *O*-methyl mandelamide derivatives (*S,R*)-**12** and (*R,R*)-**12**

The absolute configuration of alkene **9** was determined after TBAF deprotection of the sulfoximine nitrogen to give **11** followed by reaction with (*R*)-*O*-methyl mandelic acid (Scheme 4) to afford diastereomeric amides (*S*,*R*)-**12** and (*R*,*R*)-**12** in a 3:1 ratio. The ^1^H NMR resonance for the alkene signal for the alkene proton α to the sulfoximine in the major diastereoisomer of **12** occurred downfield (7.07 ppm) from that observed from the minor diastereoisomer (6.99 ppm) (a difference of +0.08 ppm) whereas the methyl group signal for the major diastereomer occurred upfield (3.26 ppm) from the methyl signal for the minor diastereoisomer (3.32 ppm) (a difference of -0.06 ppm) (Figure 2). This may be compared with the results obtained by Yabunchi and Kasumi who initially examined the NMR characteristics of 15 sulfoximine amides and rationalised the chemical shift trends on the basis of the probable favoured conformations of the amides in solution.[23]

Subsequently, this method was shown to be applicable to vinyl sulfoximines.[24] They report Δδ values which are calculated from the ^1^H NMR spectra of diastereomeric amides, prepared by coupling a single sulfoximine stereoisomer separately with both enantiomers of *O*-methyl mandelic acid. They found that the difference in chemical shift between the methyl groups of (*R*,*S*)-**13** and *S*-(*R*) isobutyl methyl *N*-[(*R*)-methoxylphenylacetyl]sulfoximine (*R*,*R*)-**13** was -0.078 ppm (the chemical shift differences must be the same for (*S*,*R*)-**13**-(*R*,*R*)-**13** which can then be compared directly with -0.06 ppm for the difference between diastereomers **12**). The differences Δδ for the protons on the methylene attached to diastereomers (*R*,*S*)-**13** and (*R*,*R*)-**13** were +0.117 ppm and +0.066 ppm which compare well with +0.08 ppm for the difference in the α vinyl protons of **12**. On this basis, the major diastereoisomer was assigned as (*S*,*R*)-**12** and therefore the minor diastereoisomer was assigned as the (*R*,*R*) isomer, indicating that the major enantiomer of **9** formed in the desymmetrisation reaction was (*S*) configured at sulfur when chiral base (*S*,*S*)-**5** was used in the presence of LiCl. This can be checked by comparison of the NMR shifts of **12** with the diastereomeric vinyl sulfoximine amides **14.**[24] For vinyl sulfoximine amides (*S*,*S*)-**14** and (*S*,*R*)-**14** δΔ for the vinylic proton was determined as -0.044 ppm, the difference (*R*,*R*)-**14** - (*R*,*S*)-**14** must represent an identity in this case, the difference in chemical shift for the depicted vinylic proton in (*R,R*)-**12** and (*S,R*)-**12** is -0.08 ppm, consistent with the configurational assignment.

**Scheme 5:**
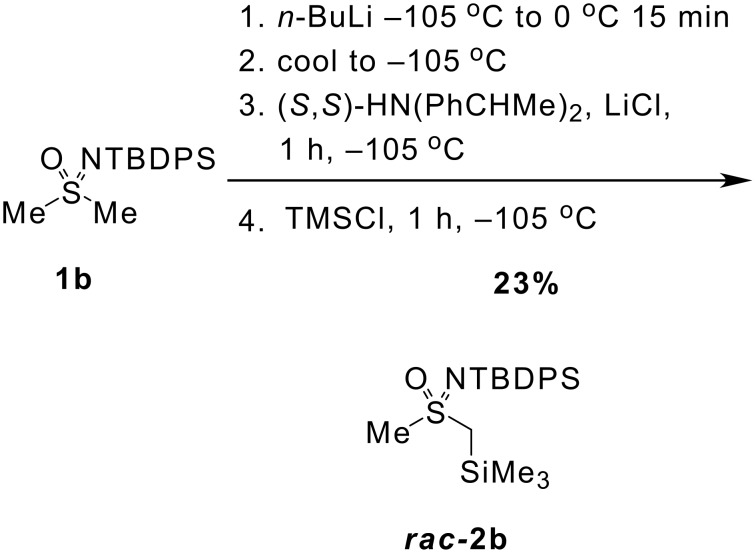
The influence of (*S,S*)-*bis*(1-phenylethyl)amine on the ee of **1b**

A mechanism involving irreversible asymmetric deprotonation to a lithiated sulfoximine followed by rapid anion quenching on addition of the electrophile appears likely. An alternative equilibration of the sulfoximine anion with the chiral amine is less probable as racemic **2b** was obtained in 23% yield after TMSCl quenching when sulfoximine **1b** was first lithiated by treatment with *n*-BuLi followed by addition of a solution of (*S*,*S*)-*bis-N,N-*(1-phenylethyl)amine and lithium chloride (Scheme 5).

In an analogous reaction, enantioenriched lithiated sulfoxides racemised via a reversible disproportionation to the sulfoxide and a symmetrical, dilithiated sulfoxide.[16] However this process seems to be unimportant for sulfoximines under the present conditions as the ee of **2g** was unchanged by reducing the deprotonation time at -105°C to 10 minutes (28%, 57% ee) or by warming from -105°C to -78°C prior to quenching (furnishing **2g** in 48% yield and 57% ee).

In contrast to the situation with most of the electrophiles studied, benzophenone and (diphenylmethylene)toluenesulfonamide may undergo a reversible addition to the sulfoximine anion, whereas in the other cases, substitution is probably irreversible and this difference may have implications for the enantioselectivity in these cases.

## Conclusion

Chiral lithium amide-mediated desymmetrisation of dimethylsulfoximines is possible. Development of more selective lithium amides should enable the realisation of truly high ee desymmetrisations. Current research efforts are directed towards this goal and towards examining the asymmetric deprotonation of other sulfoximines. We are also interested in exploring alternative, more selective desymmetrisations of dialkylsulfoximines.

## Supporting Information

File 1 The use of chiral lithium amides in the desymmetrisation of *N*-trialkylsilyl dimethyl sulfoximines. Detailed experimental procedures and characterisation data.

File 2 The use of chiral lithium amides in the desymmetrisation of *N*-trialkylsilyl dimethyl sulfoximines. ^1^H and ^13^C NMR Spectra.
