# Supplementary material for: The use of chiral lithium amides in the desymmetrisation of N-trialkylsilyl dimethyl sulfoximines
Source: Beilstein J Org Chem. 2007 Oct 16;3:33. doi: 10.1186/1860-5397-3-33 (PMC2100055; doi:10.1186/1860-5397-3-33)
Supplement: File 1 — The use of chiral lithium amides in the desymmetrisation of N-trialkylsilyl dimethyl sulfoximines. Detailed experimental procedures and characterisation data. [file Beilstein_J_Org_Chem-03-33-s001.pdf]

ADDITIONAL FILE 1

The use of chiral lithium amides in the desymmetrisation of *N*-trialkylsilyl dimethyl sulfoximines

Matthew J. McGrath and Carsten Bolm*

Institut für Organische Chemie, Landoltweg 1, RWTH Aachen, Aachen 50274, Germany.

Carsten.Bolm@oc.rwth.aachen.de

Matthew.Mcgrath@oc.rwth.aachen.de

General Experimental:

Et_2O and THF were freshly distilled from benzophenone ketyl. Pentane was distilled before use. All other solvents were used as received. (−)-Sparteine was distilled from CaH_2 before use. *N*-(diphenylmethylene)-4-toluenesulfonamide,[1] *N*-isopropyl(1-phenylethyl)amine hydrochloride[2] and 1,2-*bis*[(*S*-1-phenylethylamino]ethane dihydrochloride[3] were prepared as described in the literature. All reactions were carried out under an atmosphere of nitrogen or argon using oven dried glassware. Flash column chromatography was carried out using Silica gel 60. Thin layer chromatography was carried out using Merck F254 alumina-backed silica plates. For Kugelrohr distillation, the temperatures quoted correspond to the oven temperatures. ^1H and ^{13}C NMR spectra were recorded on Varian Innova 400 (400 or 100 MHz) or Mercury 300 (300 and 75 MHz) instruments. Chemical shifts are given in ppm and spin-spin coupling constants, *J*, are given in Hz. Melting points were determined in open-end capillary tubes on a Buchi B-540 melting point apparatus and are uncorrected. Microanalyses were obtained with a Vario EL element analyzer. Mass spectra were acquired on a Varian MAT 212 spectrometer (CI, 100 eV and EI, 70 eV) and HRMS were recorded on a Finnegan MAT 95 spectrometer. IR Spectra were taken on a Perkin-Elmer FT/IR 1760 and were recorded as KBr pellets, as the neat liquid or in a $CHCl_3$ solution. High-Performance Liquid

Chromatography was performed on a Gynkotek M480 instrument or on a Merck Hitachi Intrsument with a D7000 interface, L7100 pump and L7400 UV detector using Daicel Chiralcel OD or Chiralcel OD-H columns. All other reagents were purchased from commercial suppliers and used without further purification.

Dimethyl sulfoximine (3)

Concentrated sulfuric acid (45 mL) was cautiously added dropwise to a stirred suspension of dimethyl sulfoxide (12 mL, 169 mmol) and sodium azide (11 g, 169 mmol) in chloroform (200 mL) at 0 ^{o}C under nitrogen and the reaction was allowed to warm slowly to room temperature over 48 h. Then, a 1:1 mixture of ice and water (500 mL) was added. The layers were separated and the organic layer was washed with water (100 mL). The combined aqueous phases were neutralized with 3 M NaOH (250 mL) and concentrated under reduced pressure by rotary evaporation. The residue was triturated with ethanol (500 mL) and filtered. The filtrate was concentrated, triturated with chloroform (200 mL) and filtered. The filtrate was dried over anhydrous K_2CO_3 and concentrated to afford sulfoximine **3** (7.4 g, 52%) as a hygroscopic solid mp 53-54°C (Lit.,[4] mp 55-56 ^{o}C). ^{1}H NMR (400 MHz, $CDCl_3$) δ: 3.09 (6H , s), 2.97 (1H, s); ^{13}C NMR (100 MHz, $CDCl_3$) δ: 44.9

Spectroscopic data were consistent with those reported in the literature.[4]

***N*-(Trimethylsilyl) dimethyl sulfoximine (1a)**

Sulfoximine **3** (3.9 g, 41.9 mmol) was dissolved in hexamethyldisilazane (13.0 mL) and the resulting solution was heated to 125 ^{o}C for 2 h in a sealed tube under Ar. The solution was cooled to rt and concentrated under reduced pressure and the residue was distilled (80-105 ^{o}C, 3 mm Hg, Lit.,[5] 47 ^{o}C, 0.1 Torr) to afford trimethylsilyl derivative **1a** (4.22 g, 61%).

IR (neat) 3012, 2956, 2896, 1420, 1331, 1284, 1249, 1153, 934, 842, 756, 688, 650 cm^{-1};

^{1}H NMR (400 MHz, C_6D_6) δ: 2.20 (6H, s), 0.31 (9H, s). ^{13}C NMR (100 MHz, C_6D_6) δ: 46.9, 3.1. Spectroscopic data were consistent with the literature.[5]

_N_-(_tert_-Butyldiphenylsilyl) dimethyl sulfoximine (1b)

tert-Butyldiphenylsilyl chloride (5.0 mL, 18 mmol, 1.25 equiv.) was added dropwise to a solution of dimethylsulfoximine **3** (1.34 g, 14.4 mmol, 1.0 equiv.) and imidazole (1.96 g, 28.8 mmol, 2.0 equiv.) in DMF (8.0 mL) at 0 °C under nitrogen and the resulting solution was heated to 60 °C for 48 h. The solution was poured into water (10 mL) and extracted with CH_2Cl_2 (3 x 20 mL). The combined CH_2Cl_2 extracts were dried over $MgSO_4$ and concentrated to afford the crude product. Purification by flash chromatography (petroleum ether/ether 8:2 then CH_2Cl_2) gave TBDPS compound **1b** (3.30 g, 69%) as a white crystalline solid mp 63-65 °C. R_F 0.4 (pentane/EtOAc 4:1); IR ($CHCl_3$) 2956, 2931, 2890, 2856, 1335, 1301, 1162, 1106 cm^{-1}; ^{1}H NMR (400 MHz, C_6D_6) δ: 7.95-7.92 (4H, m), 7.28-7.22 (6H, m), 2.11 (6H, s), 1.28 (9H, s). ^{13}C NMR (100 MHz, C_6D_6) δ: 137.2, 136.1, 129.4, 127.9, 46.5, 27.6, 19.7. MS (EI) 277 (2), 276 (10), 275 (22), 274 (100, M^+-$C(CH_3)_3$), 244 (4), 212 (1), 199 (5), 181 (1), 167 (2), 135 (2), 105 (1), 77 (2). Elemental Analysis $C_{18}H_{25}NOSSi$ requires C: 65.21 H: 7.60 N: 4.22 found: C: 65.12 H: 7.56 N: 4.14.

2-Hydroxy-2,2-diphenylethyl methyl sulfoximine (2a)

n-BuLi (1.2 mL, 1.5 M in hexanes, 1.8 mmol, 1.5 equiv.) was added to a solution of (−)-sparteine **4** (418 mg, 1.78 mmol, 1.5 equiv.) in Et_2O (2.0 mL) at –78 °C under Ar. Then, a solution of sulfoximine **1a** (202 mg, 1.22 mmol, 1.0 equiv.) in Et_2O (3.0 mL) was added dropwise and the resulting solution was stirred for 2 h at −78 °C. A solution of benzophenone (387 mg, 2.12 mmol, 1.7 equiv.) in Et_2O (12 mL) was added and the resulting solution was allowed to warm to rt slowly over 18 h. The reaction was quenched by addition of 1M HCl (5.0 mL) and extracted with EtOAc (3 x 10 mL). The aqueous phase was neutralised with

saturated aqueous Na_2CO_3 (0.5 mL) and extracted with EtOAc (3 x 10 mL). The combined organic layers were dried over $MgSO_4$ and concentrated. The residue was subjected to flash chromatography to afford **2a** (156 mg, 41%) as a white solid, mp 106-107 ^{o}C. HPLC: Chiralcel OD, heptane/*i*-PrOH 70:30, 0.5 mL•min^{-1}, 14.6 min (minor), 16.5 min (major), 8% ee; R_F 0.5 (99:1 CH_2Cl_2/MeOH); IR (KBr, cm^{-1}) 3451, 3424, 1596, 1492, 1449, 1410, 1320, 1216, 1061, 1017, 843, 780, 755, 697, 600. ^{1}H NMR (400 MHz, $CDCl_3$) δ: 7.52-7.26 (12H, m), 4.03 (1H, d, 14 Hz), 3.99 (1H, d, 14 Hz), 2.42 (3H, s). ^{13}C NMR (100 MHz, $CDCl_3$) δ: 144.7, 143.9, 128.4, 128.3, 127.6, 127.5, 126.1, 125.7, 75.5, 64.2, 45.8. Elemental analysis $C_{15}H_{17}NO_2S$ requires C: 65.45 H: 6.18 N: 5.09 found: C: 65.38 H: 6.52 N: 4.90.

General Procedure A: Lithiation-Electrophilic trapping of sulfoximine 1b with chiral lithium amide 5 at –105 ^{o}C

n-BuLi (1.2 mL, 1.74 mmol, 2.7 equiv.) was added dropwise with stirring to a suspension of (*S,S*)-*bis*(1-phenylethyl)amine hydrochloride (253 mg, 0.967 mmol, 1.5 equiv.) in THF (5.0 mL) at –105 ^{o}C. The resulting solution was allowed to warm to RT and was kept at that temperature for 20 min before being recooled to –105 ^{o}C. A solution of sulfoximine **1b** (213 mg, 0.694 mmol, 1.0 equiv.) in THF (3.0 mL) was then added via syringe and the resulting solution was maintained at –105 ^{o}C for a further hour. Then, a solution of electrophile (1.0 equiv., 2.0 eq.) in THF (5.0 mL) was added and the resulting solution was stirred for 30 minutes. The reaction was quenched by addition of saturated aqueous $NaHCO_3$ (5 mL) and extracted with ether (3 x 20 mL). The ether layers were combined and washed with 5% aqueous H_3PO_4 (2 x 20 mL) and saturated aqueous $NaHCO_3$ (20 mL), dried over $MgSO_4$ and concentrated. The resulting product was purified by flash column chromatography.

***N*-(*tert*-Butyldiphenylsilyl) methyl trimethylsilylmethyl sulfoximine (2b)**

Using **general procedure A**, *n*-BuLi (1.2 mL, 1.73 mmol, 1.4 M in hexanes, 2.7 equiv.), (*S,S*)-*bis*(1-phenylethyl)amine hydrochloride (252 mg, 0.964 mmol, 1.5 equiv.), sulfoximine **1b** (212 mg, 0.642 mmol, 1.0 equiv.) and TMSCl (0.105 mL, 0.843 mmol., 1.3 equiv.) in THF (11 mL) gave the crude product. Purification by flash chromatography (9% ethyl acetate/pentane) afforded silyl adduct **2b** as a white solid (152 mg, 58%), mp 78-79 °C, $[\alpha]_D$ -25.4 (c, 0.31, $CHCl_3$). HPLC: Chiralcel OD-H, heptane/*i*-PrOH, 99:1, 0.5 mL•min^{-1}, 254 nm, t_{R1}18 min (minor) t_{R2} 29 min (major), 61% ee; R_F 0.6 (EtOAc/petroleum ether); IR (KBr) 2957, 2930, 2897, 2852, 1472, 1428, 1312, 1274, 1154, 1104, 965, 851, 783, 744, 703, 637, 588, 493 cm^{-1}; ^{1}H NMR (400 MHz, C_6D_6) δ: 8.04-8.02 (2H, m), 7.94-7.92 (2H, m), 7.30-7.21 (6H, m), 2.23 (1H, d, 13 Hz), 2.15 (3H, s), 2.13 (1H, d, 13 Hz), 1.29 (9H, s), 0.18 (9H, s); ^{13}C NMR (100 MHz, C_6D_6) δ: 137.4, 137.2, 136.4, 136.2, 129.4, 129.3,127.9, 127.8, 50.9, 48.6, 27.8, 19.8, 0.2; Elemental Analysis $C_{21}H_{33}NOSi_2S$ requires: C: 62.51 H: 8.18 N: 3.47 found: C: 62.66 H: 8.22 N: 3.44.

***N*-(*tert*-Butyldiphenylsilyl) 2-hydroxy-2,2-diphenylethyl methyl sulfoximine (2c)**

Using **general procedure A**, *n*-BuLi (1.2 mL, 1.74 mmol, 2.7 equiv.), (*S,S*)-*bis*-(1-phenylethyl)amine hydrochloride (253 mg, 0.967 mmol, 1.5 equiv.), sulfoximine **1b** (213 mg, 0.694 mmol, 1.0 equiv.), and benzophenone (152 mg, 0.837 mmol, 1.0 equiv.), in THF (13.0 mL) gave the crude product. The crude product was purified by flash chromatography (10:1 petrol ether/ethyl acetate) to afford compound **2c** as a white solid (284 mg, 86% yield), mp 160-162 °C, $[\alpha]_D$ +5.1 (c, 0.3, $CHCl_3$). HPLC: Chiralcel OD-H, heptane: *i*-PrOH 99:1, 0.5 mL.min^{-1}, 254 nm, t_{R1} 39 min (major), t_{R2} 59 min (minor) 70% ee; R_F 0.2 (10:1 petroleum ether/EtOAc); IR (KBr) 3450, 2964, 2928, 2851, 1701, 1451, 1427, 1366, 1325, 1293, 1262, 1139, 1104, 1063, 977, 819, 785, 743, 702, 606, 500 cm^{-1}; ^{1}H NMR (400 MHz, C_6D_6) δ: 7.73-7.68 (4H, m), 7.36 (2H, d, 7 Hz), 7.27 (2H, d, 7 Hz), 7.11-7.05 (6H, m), 7.05-6.83 (6H, m), 3.65 (1H, d, 14 Hz), 3.57 (1H, d, 14 Hz), 1.72 (3H, s), 1.24 (9H, s); ^{13}C NMR (100 MHz,

$CDCl_3$) δ: 145.4, 145.3, 135.9, 135.9, 135.8, 135.9, 129.3, 129.2, 75.9, 66.0, 46.8, 27.1, 27.0, 19.2; MS (CI, CH_4) 516 (7), 515 (24), 514 (60, M+H), 456 (18), 392 (13), 365 (23), 364 (100), 316 (41), 276 (24), 246 (25); HRMS: $C_{31}H_{35}NO_2SSi$ –C_4H_9 requires 456.145356 measured: 456.145530 .

_N_-(_tert_-Butyldiphenylsilyl) methyl 2-(4-toluenesulfonamido)-2,2-diphenylethyl sulfoximine (2d)

Using **general procedure A** *n*-BuLi (1.35 mL, 1.88 mmol, 2.7 equiv.) (*S*,*S*) -*bis*-(1-phenylethyl)amine hydrochloride (270 mg, 1.02 mmol, 1.5 equiv.), sulfoximine **1b** (231 mg, 0.698 mmol) and *N*-(diphenylmethylene)-4-toluenesulfonamide (287 mg, 0.738 mmol, 1.2 equiv.) in THF (15.0 mL) gave the crude product. The residue was purified by flash chromatography (9:1 then 8:1 pentane:ethyl acetate) to afford amine **2d** as a white solid (139 mg, 30%) mp 154-156 ^oC, $[\alpha]_D$ –14.7 (c, 0.17, $CHCl_3$). HPLC: Chiralcel OD-H, heptane/i-PrOH 99:1, 0.5 mL•min^{-1}, 254 nm, t_{R1} 73 min (major), t_{R2} 97 min (minor), 28% ee; R_F 0.6 (4:1 pentane/ethyl acetate); IR (KBr): 3916, 3787, 3437, 2960, 2849, 2374, 1629, 1333, 1263, 1150, 906, 816, 701, 552, 488 cm^{-1}; ^{1}H NMR (300 MHz, $CDCl_3$) δ: 7.95 (1H, s), 7.76-7.67 (4H, m), 7.40-6.94 (20H, m), 3.88 (1H, d, 15 Hz), 3.81 (1H, d, 15 Hz), 2.33 (3H, s), 1.78 (3H, s), 1.11 (9H, s); ^{13}C NMR (75MHz, $CDCl_3$) δ: 142.0, 140.1, 139.3, 139.3, 135.8, 129.3, 129.2, 129.2, 128.9, 128.0, 127.9, 127.8, 127.6, 127.5, 126.8, 68.7, 64.9, 46.6, 27.1, 21.4, 19.1; MS (CI, CH_4) 668 (4, M+2), 667 (8, M+1), 438 (8), 418 (11), 350 (21), 332 (18), 256 (15), 240 (21), 224 (33), 196 (35), 172 (100), 155 (28), 103 (50); Elemental Analysis $C_{38}H_{42}N_2O_3S_2Si$ requires C: 68.43, H 6.35, N. 4.20 found: C: 68.76 H: 6.54 N: 4.02

_N_-(_tert_-Butyldiphenylsilyl) methyl phenylthiomethylene sulfoximine (2e)

Using **general procedure A**, *n*-BuLi (1.5 mL, 2.1 mmol, 1.4 M in hexanes, 2.7 equiv.), sulfoximine **1b** (258 mg, 0.778 mmol, 1.0 equiv.), (*S*,*S*)-*bis*-(1-phenylethyl)amine

hydrochloride (305 mg, 1.17 mmol, 2.7 equiv.) and diphenyl disulfide (220 mg, 1.07 mmol., 1.3 equiv.) in THF (14 mL) gave the crude product. Purification by flash chromatography (10:1 petroleum ether/ethyl acetate) afforded sulfide **2e** as a white solid (22 mg, 6%) mp 100-101 ^{o}C, $[\alpha]_D$ +22.5 (c, 1.56, $CHCl_3$). HPLC: Chiralcel OD-H, heptane/*i*-PrOH, 99:1, 0.5 mL•min^{-1}, 254 nm, t_{R1} = 36 min (major), t_{R2} = 40 min (minor), 62% ee; R_F 0.8 (pentane/EtOAc 4:1); IR ($CHCl_3$) 2927, 2855, 1583, 1476, 1428, 1291, 1167, 1109, 968, 839, 706, 634, 604 cm^{-1}; ^{1}H NMR (300 MHz, C_6D_6) δ: 7.96-7.90 (4H, m), 7.25-7.15 (8H, m), 6.92-6.84 (3H, m), 3.73 (1H, d 14 Hz), 3.64 (1H, d 14 Hz), 2.21 (3H, s), 1.26 (9H, s); ^{13}C NMR (100 MHz, C_6D_6) δ: 136.9, 136.8, 136.2, 136.1, 134.5, 130.8, 129.5, 129.5, 129.3, 127.9, 127.9, 127.5, 61.1, 42.7, 27.6, 19.8; MS (EI) 384 (15), 383 (27), 382 (100, M^+-$C(CH_3)_3$), 292 (16), 291 (64), 245 (6), 244 (4), 213 (9), 199 (6), 197 (2); HRMS: $C_{24}H_{29}NOS_2Si - C_4H_9$ requires 382.075564 measured 382.075711.

***N*-(*tert*-Butyldiphenylsilyl) 3-butenyl methyl sulfoximine (2f)**

Using **general procedure A**, *n*-BuLi (2.3 mL, 1.35 M solution, 3.13 mmol, 2.7 equiv.), sulfoximine **1b** (385 mg, 1.16 mmol, 1.0 equiv.), (*S*,*S*)-*bis*(1-phenylethyl)amine hydrochloride (456 mg. 1.74 mmol, 1.5 equiv.) and allyl iodide (0.24 mL, 2.57 mmol, 2.2 equiv.) in THF (12 mL) gave the crude product. Purification by flash chromatography (10% ethyl acetate/pentane) gave compound **2f** as a yellow oil (148 mg, 34%), $[\alpha]_D$ +5.4 (c, 0.10, $CHCl_3$);. R_F 0.8 (4:1, pentane/EtOAc); IR ($CHCl_3$ cm^{-1}) 3069, 3012, 2932, 2892, 2857, 1642, 1472, 1427, 1300, 1152, 1107, 999, 922, 822, 751, 703, 649, 600, 503; ^{1}H NMR (300 MHz, C_6D_6) δ: 7.99-7.92 (4H, m), (6H, m), 5.44-5.31 (1H, m), 4.83-4.74 (2H, m), 2.52-2.47 (2H, m), 2.31-2.23 (2H, m), 2.09 (3H, s), 1.29 (9H, s); ^{13}C NMR (75 MHz, $CDCl_3$) 136.5, 136.3, 135.6, 135.5, 134.5, 129.1, 127.4, 116.8, 57.8, 44.7, 27.6, 27.1, 19.2; MS (EI) 316 (11), 315 (27), 314 (100, M^+ - 57 (-$C(CH_3)_3$), 287 (10), 286 (45), 260 (14), 212 (19), 199 (22); HRMS: $C_{21}H_{29}NOSSi - C_4H_9$ requires 314.103491 measured: 314.103478.

***N*-(*tert*-Butyldiphenylsilyl) 2-bromophenylethyl methyl sulfoximine (2g)**

Using **general procedure A**, *n*-BuLi (1.2 mL, 1.43 M in hexanes, 1.74 mmol, 2.7 equiv.), (*S,S*)-(1-phenylethyl)amine hydrochloride (253 mg, 0.967 mmol, 1.5 equiv.), sulfoximine **1b** (213, 0.645 mmol, 1.0 equiv.), and 2-bromobenzyl bromide (400 mg, 1.84 mmol, 2.3 equiv.) gave the crude product. Purfication by flash chromatography (10% then 20% ethyl acetate/pentane) gave the title compound as a colorless oil (223 mg, 70%), $[\alpha]_D$ –7.7 (c, 1.7, $CHCl_3$). HPLC: Chiralcel OD-H, 99:1 heptane/iPrOH, 0.5 mL•min^{-1}, 230 nm, t_{R1} 51 min (major), t_{R2} 92 min (minor), 57% ee; R_F 0.7 (pentane/EtOAc 3:1); IR ($CHCl_3$): 3067, 3014, 2931, 2890, 2855, 1472, 1427, 1299, 1158, 1107, 1025, 941, 822, 753, 704, 640, 600, 503 cm^{-1}; ^{1}H NMR (400 MHz, $CDCl_3$) δ: 7.80-7.76 (4H, m), 7.53 (1H, dd, 8, 1 Hz), 7.40-7.37 (7H, m), 7.25-7.21 (1H, m), 7.11 (1H, t, 7 Hz), 3.22-3.14 (4H, m), 2.73 (3H, s), 1.11 (9H, s); ^{13}C NMR (100 MHz, $CDCl_3$) δ: 137.5, 135.64, 135, 58, 130.7, 128.6, 127.9, 58.1, 44.5, 30.6, 22.1, 18.3; MS (EI, DP): *m/z* (%) = 444 (100, M^+ (^{81}Br)- $C(CH_3)_3$), 442 (96, M^+ (^{79}Br)-$C(CH_3)_3$), 366 (18), 364 (15), 212 (8), 199 (23), 104 (9); HRMS: $C_{25}H_{30}NO^{79}BrSSi$-C_4H_9 requires 442.029664 measured 442.029693

General Procedure B Preparation of Vinyl Sulfoximines

n-BuLi (2.7 equiv.) was added to a suspension of bis(1-phenylethyl)amine hydrochloride (1.25 mmol, 1.5 equiv.) in THF (5.0 mL) at –105 °C, the resulting resulting mixture was allowed to warm to room temperature slowly over 30 minutes after which the salt had completely dissolved and the solution had attained a yellow colour. The solution was recooled to –105 °C at which time it became purple. A solution of sulfoximine **1b** (276 mg, 0.834 mmol, 1.0 equiv.) in THF (5.0 mL) was added dropwise over 5 minutes and the solution was kept at –105 °C for a further 1 h after which diethyl chlorophosphate (1.3 equiv.) was added.

After five minutes, a solution of *t*-BuOK (1.3 equiv.) was added and the resulting yellow solution was stirred for a further 10 min after which a solution of aldehyde (1.7 equiv.) in THF (3.0 mL) was added. The solution was warmed to 0 ^{o}C over 1 h and quenched with 5% aqueous H_3PO_4 (20 mL). The reaction mixture was extracted with Et_2O (3 x 20 mL) and washed with 5% aqueous H_3PO_4 (2 x 20 mL). The combined organic layers were dried over $MgSO_4$ and concentrated. The product was purified by flash chromatography.

***N-(tert*-Butyldiphenylsilyl) 4-chlorostyryl methyl sulfoximine (9)**

Using **general procedure B** *n*-BuLi (1.6 mL, 1.4 M solution in THF, 2.7 equiv.), (*S*,*S*)-*bis*(1-phenylethyl)amine hydrochloride (327 mg, 1.25 mmol, 1.5 equiv.), sulfoximine **1b** (276 mg, 0.834 mmol, 1.0 equiv.) diethyl chlorophosphate (0.16 mL, 1.08 mmol, 1.3 equiv.), *t*-BuOK in THF (1.6 mL, 0.68M in THF, 1.3 equiv.) and 4-chlorobenzaldehyde (204 mg, 1.45 mmol, 1.7 equiv.) in THF (13 mL) gave the crude product. Purification by flash chromatography (10% ethyl acetate/pentane then 20% ethyl acetate/pentane) gave adduct **9** (198 mg, 52%) as a colourless oil, $[\alpha]_D$ -0.55 (c, 0.1, $CHCl_3$).; HPLC; Chiralcel OD-H, heptane/i-PrOH 99:1, 254 nm, 0.5 mL•min^{-1}, t_{R1} 30.4 min (major), t_{R2} 39.4 min (minor), 55% ee; R_F 0.6 (pentane/EtOAc 4:1); IR ($CHCl_3$): v = 3049, 3017, 2932, 2890, 2856, 1621, 1593, 1490, 1426, 1303, 1150, 1105, 1010, 970, 810, 757, 704, 646, 601, 501 cm^{-1}; ^{1}H NMR (400 MHz, $CDCl_3$) δ: 7.77-7.73 (4H, m), 7.37-7.30 (9H, m), 7.19 (2H, d, 8 Hz) 6.71 (1H, d, 15 Hz), 2.00 (3H, s), 1.11 (9H, s); ^{13}C NMR (100 MHz, $CDCl_3$) δ: 138.7, 136.2, 136.2, 135.5, 131.2, 129.3, 129.0, 127.4, 47.7, 27.2, 19.4; MS (EI, DP) = 398 (31, (M+2)-C(CH_3)$_3$), 396 (100, M+ - C(CH_3)$_3$), 332 (3), 306 (2), 245 (14), 241 (6), 199 (7); Elemental Analysis $C_{25}H_{28}SONSiCl$ requires C: 66.12 H: 6.22 N: 3.08 found: C: 65.73 H: 6.38 N: 3.24.

***N*-(*tert*-Butyldiphenylsilyl) methyl 4-phenylstyryl sulfoximine (10)**

Using **general procedure B** *n*-BuLi (1.7 mL, 1.45 M solution in THF, 2.47 mmol, 2.7 equiv.) (*S,S*)-*bis*(1-phenylethyl)amine hydrochloride (358 mg, 1.37 mmol, 1.5 equiv.), sulfoximine **1b** (304 mg, 0.913 mmol, 1.0 equiv.), diethyl chlorophosphate (0.16 mL, 1.10 mmol, 1.3 equiv.), *t*-BuOK (0.75 mL, 1.45 M in THF, 1.3 equiv.) and biphenyl 4-carboxaldehyde (217 mg, 1.19 mmol, 1.3 equiv.) in THF (13 mL) gave the crude product. Purification by flash chromatography (10% ethyl acetate/pentane then 20% ethyl acetate/pentane) gave the title compound (208 mg, 46%) as a colourless oil, $[\alpha]_D$ -0.83 (c, 0.3, $CHCl_3$). HPLC: Chiralcel OD-H, heptane/*i*-PrOH 99:1, 0.5 mL•min^{-1}, 254 nm, 66.9 min (major), 87.9 min (minor), 52% ee. R_F 0.1 (9:1 petroleum ether/EtOAc); IR ($CHCl_3$) v 3066, 3050, 2955, 2932, 2856, 1674, 1609, 1485, 1300, 1149, 1107, 970, 816, 760, 701 cm^{-1}; ^{1}H NMR (400 MHz, C_6D_6) δ: 8.05-8.01 (4H, m), 7.52 (1H, d, 15 Hz), 7.40-7.37 (2H, m), 7.30-7.19 (10H, m), 7.15-7.10 (1H, m) 6.94 (2H, d, 8 Hz), 6.56 (1H, d, 15 Hz), 2.43 (3H, s), 1.36 (9H, s); ^{13}C NMR (75 MHz, $CDCl_3$) δ: 143.1, 139.9, 139.7, 136.4, 135.6, 131.7, 130.6, 129.0, 128.9, 128.7, 127.9, 127.4, 127.4, 127.0, 47.8, 27.1, 19.3; MS (CI, isobutene) 498 (4 ,M^++3), 497 (10, M^++2), 496 (24,M^++1), 439 (4), 438 (13), 419 (33), 418 (100), 358 (2), 183 (2); HRMS $C_{31}H_{33}NOSSi$ – C_4H_9 requires: 438.134791 measured: 438.134879.

***N-H* Methyl 4-chlorostyryl sulfoximine (11)**

TBAF (0.6 mL, 0.6 mmol, 1.0 M solution in THF, 2.0 equiv.) was added to a solution of TBDPS derivative **9** (142 mg, 0.31 mmol, 1.0 equiv.) in THF (5.0 mL) under nitrogen and the resulting solution was stirred at rt for 48 h. The solution was concentrated and the residue was purified by flash column chromatography (EtOAc) to afford sulfoximine **11** (49 mg, 73%) as an oil that solidified on standing, mp 120 ^{o}C. R_F 0.2 (EtOAc); IR($CHCl_3$) 3325, 3023, 2924, 1619, 1589, 1490, 1405, 1321, 1285, 1247, 1201, 1093, 1012, 932, 852, 819, 787, 732, 665 cm^{-1}; ^{1}H NMR (300 MHz, $CDCl_3$) δ: 7.54 (1H, d, 15 Hz), 7.46 (2H, d, 8.5 Hz), 7.39 (2H, d, 8.5 Hz), 7.01 (1H, d 15 Hz), 3.10 (3H, s), 2.29 (1H, br s); ^{13}C NMR (75 MHz, $CDCl_3$) δ:

141.1, 137.1, 130.9, 130.1, 129.6, 129.4, 44.9. HRMS $C_9H_{10}NOSCl$ –CH_3OS requires: 152.026702 measured: 152.026762.

_S_-(_S_)-methyl 4-chlorostyryl _N_-[(_R_)-methoxyphenylacetyl]sulfoximine [(_S_,_R_)-12] and _S_-(_R_)- methyl 4-chlorostyryl N-[(_R_)-methoxyphenylacetyl]sulfoximine [(_R_,_R_)-12]

A solution of sulfoximine **11** (38 mg, 0.176 mmol, 1.0 equiv.), (*R*)-*O*-methyl mandelic acid (61 mg, 0.353 mmol, 2.0 equiv.), HOBT•H_2O (54 mg, 0.353 mmol, 2.0 equiv.), bromo-*tris*-pyrrolidinophosphonium hexafluorophosphate (143 mg, 0.353 mmol, 2.0 equiv.) and pyridine (0.2 mL) in CH_2Cl_2 (2.0 mL) was stirred at room temperature over night. After which, EtOAc (10 mL) was added and the solution was washed with 1M HCl (10 mL), saturated aqueous $NaHCO_3$ (10 mL) and brine (10 mL), dried over $MgSO_4$ and concentrated. Purification by flash column chromatography (1:1 pentane/ethyl acetate) gave a ca 3:1 mixture of two diastereomeric amides (*S*,*R*)-**12** and (*R*,*R*)-**12** (43 mg, 67%) as a colourless oil. R_F 0.25 (1:1 EtOAc/petroleum ether); IR ($CHCl_3$) 3016, 2929, 2825, 1643, 1492, 1453, 1406, 1218, 1100, 1021, 980, 858, 752 cm^{-1}; ^1H NMR (300 MHz, $CDCl_3$) δ: (*S,R*), (*R,R*) in a 3:1 ratio 7.56-7.32 (10H, m, (*S,R*)), 7.56-7.32 (10H, m, (*R*,*R*)), 7.08 (1H, d, 15 Hz, (*S,R*) S*CH*=), 6.99 (1H, d, 15 Hz, (*R,R*) SC*H*=), 4.77(1H, s, (*R,R*) CHOMe), 4.76 (1H, s, (*S,R*) *CH*OMe), 3.41 (3H, s, (*R,R*)-O*Me*), 3.40 (3H, s, (*S*,*R*)-O*Me*), 3.31 (3H, s, (*R,R*) S*Me*), 3.26 (3H, s, (*S,R*) *SMe*); ^{13}C NMR (75 MHz, $CDCl_3$) δ: 179.1, 143.3, 137.8, 137.6, 130.2, 129.9, 129.5, 128.4, 128.2, 127.3, 123.8, 123.7, 85.7, 57.1, 42.3, 42.1 (not all of the expected carbon signals for each diastereomer are resolved); MS (EI, DP) = 244 (36, M^+ (^{37}Cl) – CHOMePh), 242 (100, M^+ (^{35}Cl) – CHOMePh), 137 (11), 121 (30), 102 (4), 91 (4), 77 (6); HRMS: $C_{18}H_{18}NO_3{}^{35}ClS$ – C_8H_9O requires 242.04254 measured 242.004463.

Experiment to probe the Reversibility of Sulfoximine Deprotonation _N_-(_tert_-Butyl-diphenylsilyl) methyl trimethylsilylmethyl sulfoximine (2b)

Sulfoximine **1b** (463 mg, 1.4 mmol, 1.0 equiv.) was dissolved in THF (10 mL) and the resulting solution was cooled to −105 °C. *n*-BuLi (1.1 mL of a 1.4 M solution in hexanes, 1.54 mmol. 1.1 equiv.) was added and the resulting solution was allowed to warm to 0 °C over 15 minutes. The solution was recooled to −105 °C and a solution of (*S,S*)-*bis*-(1-phenylethyl)amine (450 mg, 2 mmol, 1.5 equiv.) and lithium chloride (89 mg, 2 mmol, 1.5 equiv.) in THF (10 mL) was added dropwise via syringe over 5 minutes. The resulting solution was stirred at −105 °C for 30 minutes and then quenched by addition of TMSCl (0.3 mL, 2.3 mmol). The solution was allowed to warm to room temperature over night and was then quenched by addition of 5% aqueous H_3PO_4 (20 mL), extracted with ether (3 x 20 mL) and washed successively with 5% aqueous H_3PO_4 (2 x 20 mL) and saturated aqueous $NaHCO_3$ (20 mL). The combined organic layers were dried over $MgSO_4$ and concentrated and the residue was purified by flash chromatography (10:1 pentane/EtOAc) to give **2b** as a white solid (128 mg, 23%). HPLC: Chiral HPLC: Chiralcel OD-H, heptane/*i*-PrOH, 99:1, 0.5 mL•min^{-1}, 254 nm, t_{R1} 18 min t_{R2} 29 min, 0% ee. Spectroscopic data were consistent with those given above.

References:

1. Ram RN, Khan AA: *Synth Commun.* 2001, **31**,841-846.

2. Cain CM, Cousins RPC, Coumbarides G, Simpkins NS: *Tetrahedron.* 1990, **46**:523-544.

3. Fanizzi FP, Maresca L, Natile G, Lanfranchi M, Manotti-Lanfredi AM, Tiripicchio A: *Inorg Chem.* 1988, **27**: 2422-2431.

4. Schmidbaur H, Kammel, G: *Chem Ber.* 1969, 4128-4135.

5. Iley J, Bassindale AR, Patel P: *J Chem Soc Perkin 2*, 1984, 77-80.
