# Supplementary material for: The use of chiral lithium amides in the desymmetrisation of N-trialkylsilyl dimethyl sulfoximines
Source: Beilstein J Org Chem. 2007 Oct 16;3:33. doi: 10.1186/1860-5397-3-33 (PMC2100055; doi:10.1186/1860-5397-3-33)
Supplement: File 2 — The use of chiral lithium amides in the desymmetrisation of N-trialkylsilyl dimethyl sulfoximines. 1H and 13C NMR Spectra. [file Beilstein_J_Org_Chem-03-33-s002.pdf]

ADDITIONAL FILE 2

The use of chiral lithium amides in the desymmetrisation of *N*-trialkylsilyl dimethyl sulfoximines

Matthew J. McGrath and Carsten Bolm*

Institut für Organische Chemie, Landoltweg 1, RWTH Aachen, Aachen 52074, Germany.

Carsten.Bolm@oc.rwth.aachen.de

Matthew.Mcgrath@oc.rwth.aachen.de

^{1}H and ^{13}C NMR Spectra.

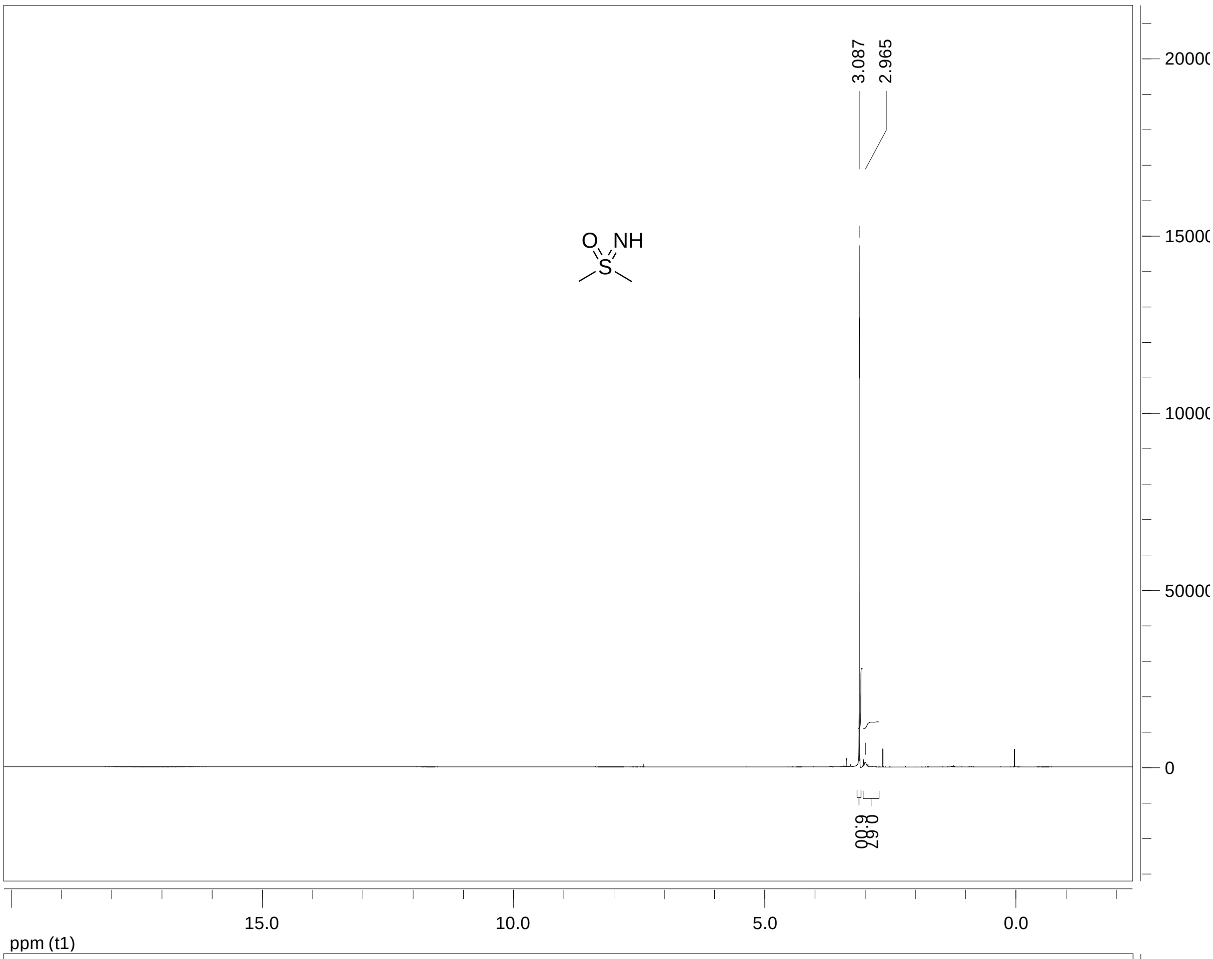
3.087
2.965
O NH
S
6.00
0.62
15.0
10.0
5.0
0.0
ppm (t1)

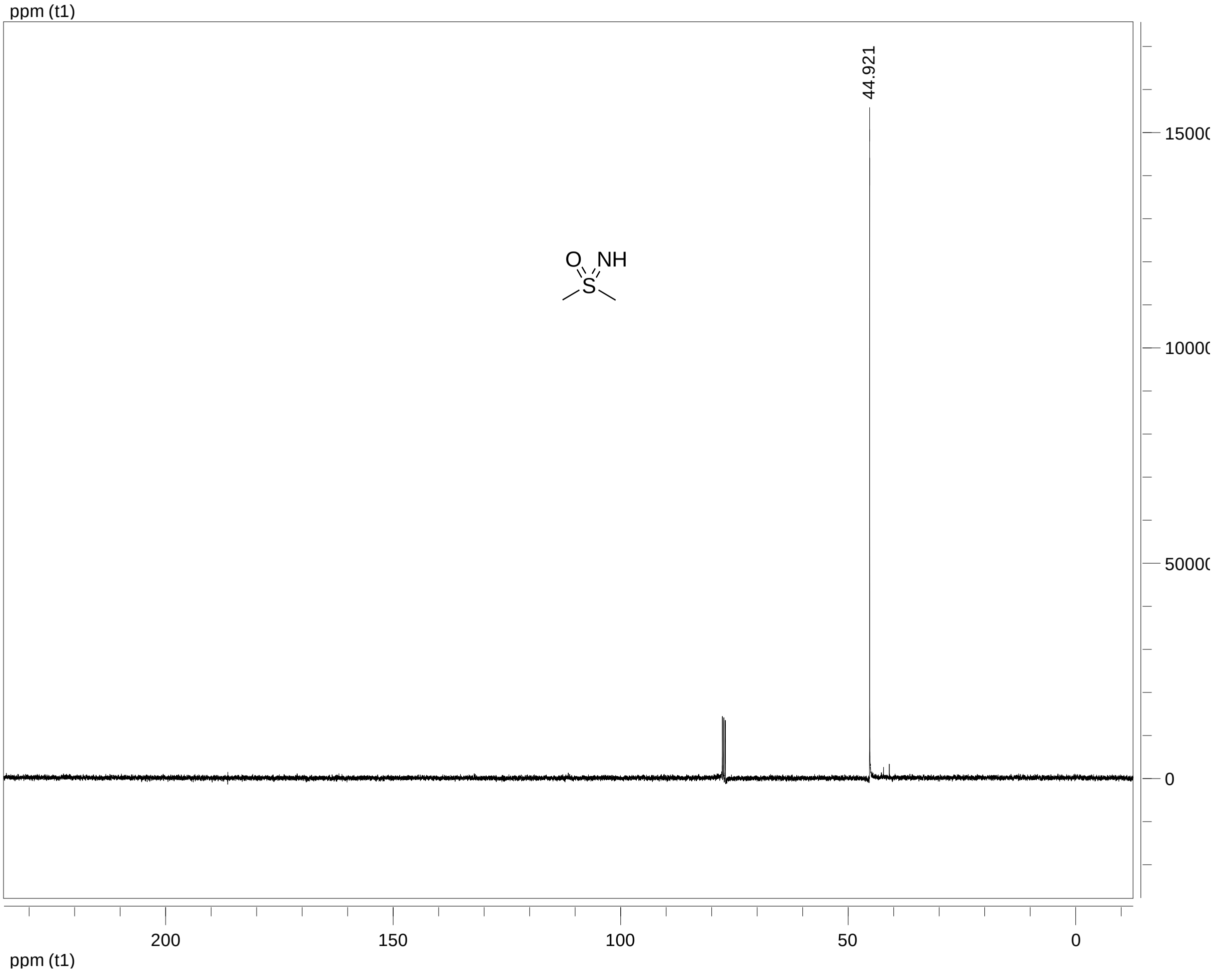
44.921
O NH
S
200
150
100
50
0
ppm (t1)

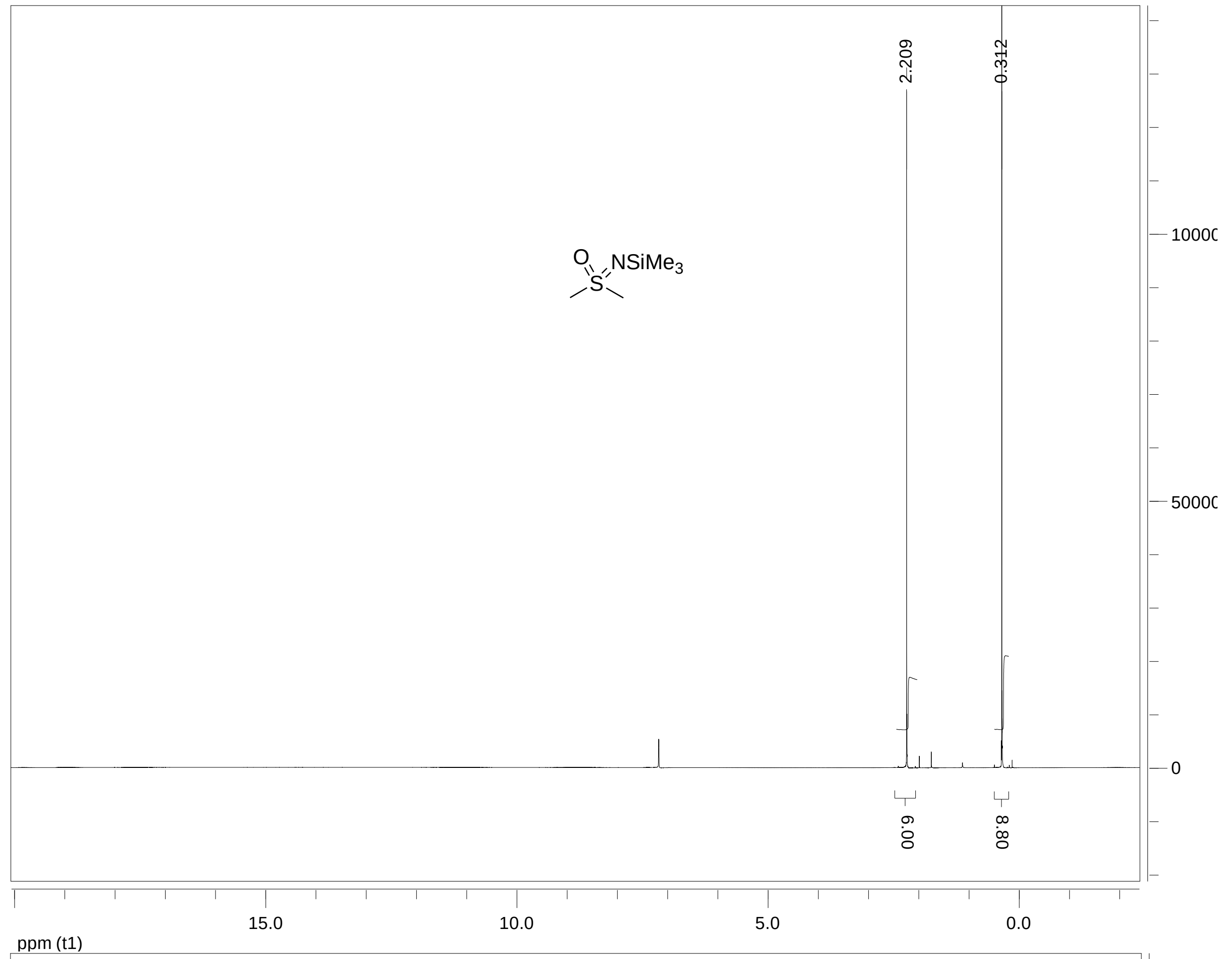
2.209
0.312
NSiMe3
6.00
8.80
15.0
10.0
5.0
0.0
ppm (t1)

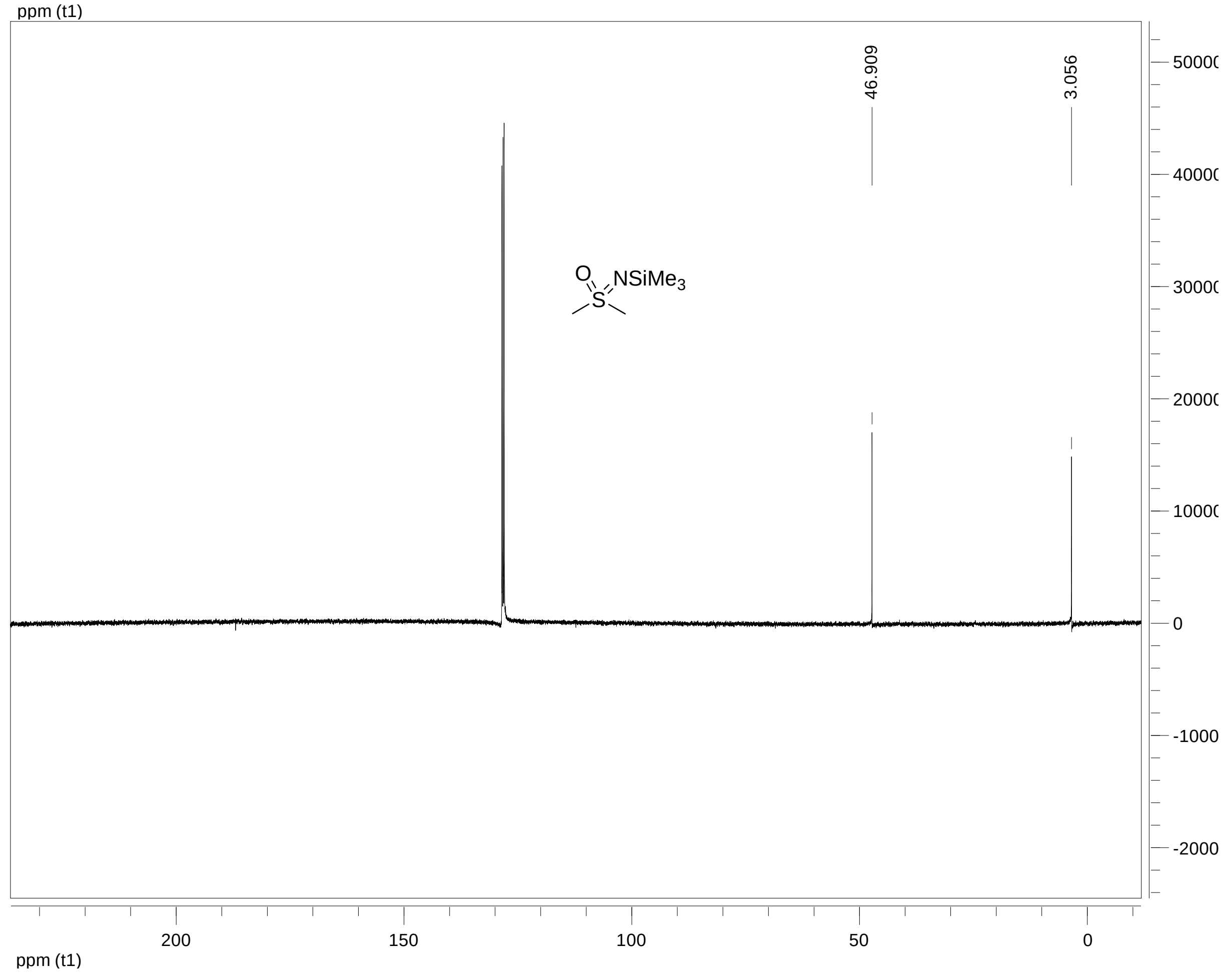
46.909
3.056
NSiMe3
200
150
100
50
0
ppm (t1)

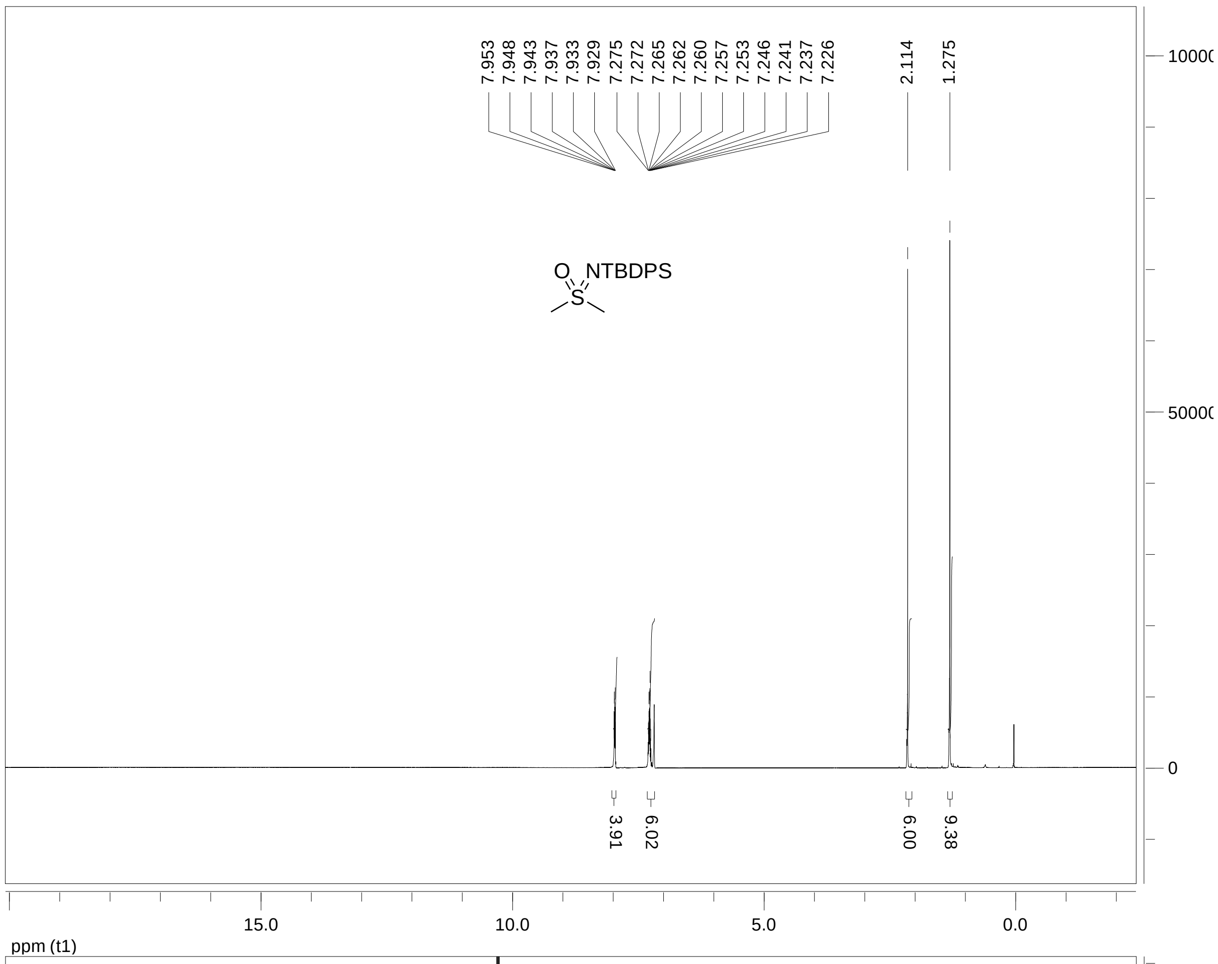

7.953
7.948
7.943
7.937
7.933
7.929
7.275
7.272
7.265
7.262
7.260
7.257
7.253
7.246
7.241
7.237
7.226
2.114
1.275
NTBDPS
3.91
6.02
6.00
9.38
15.0
10.0
5.0
0.0
ppm (t1)

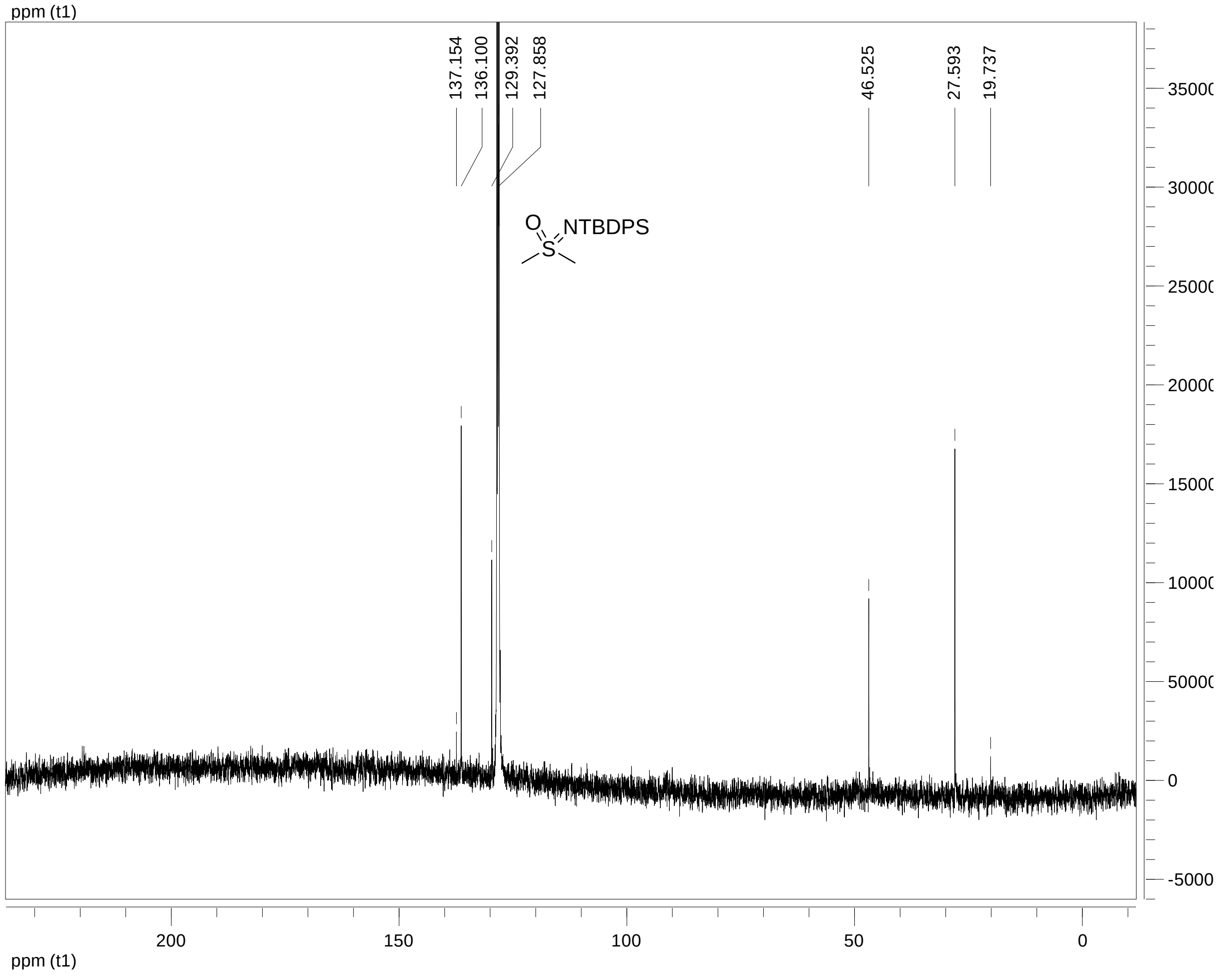

137.154
136.100
129.392
127.858
46.525
27.593
19.737
NTBDPS
200
150
100
50
0
ppm (t1)

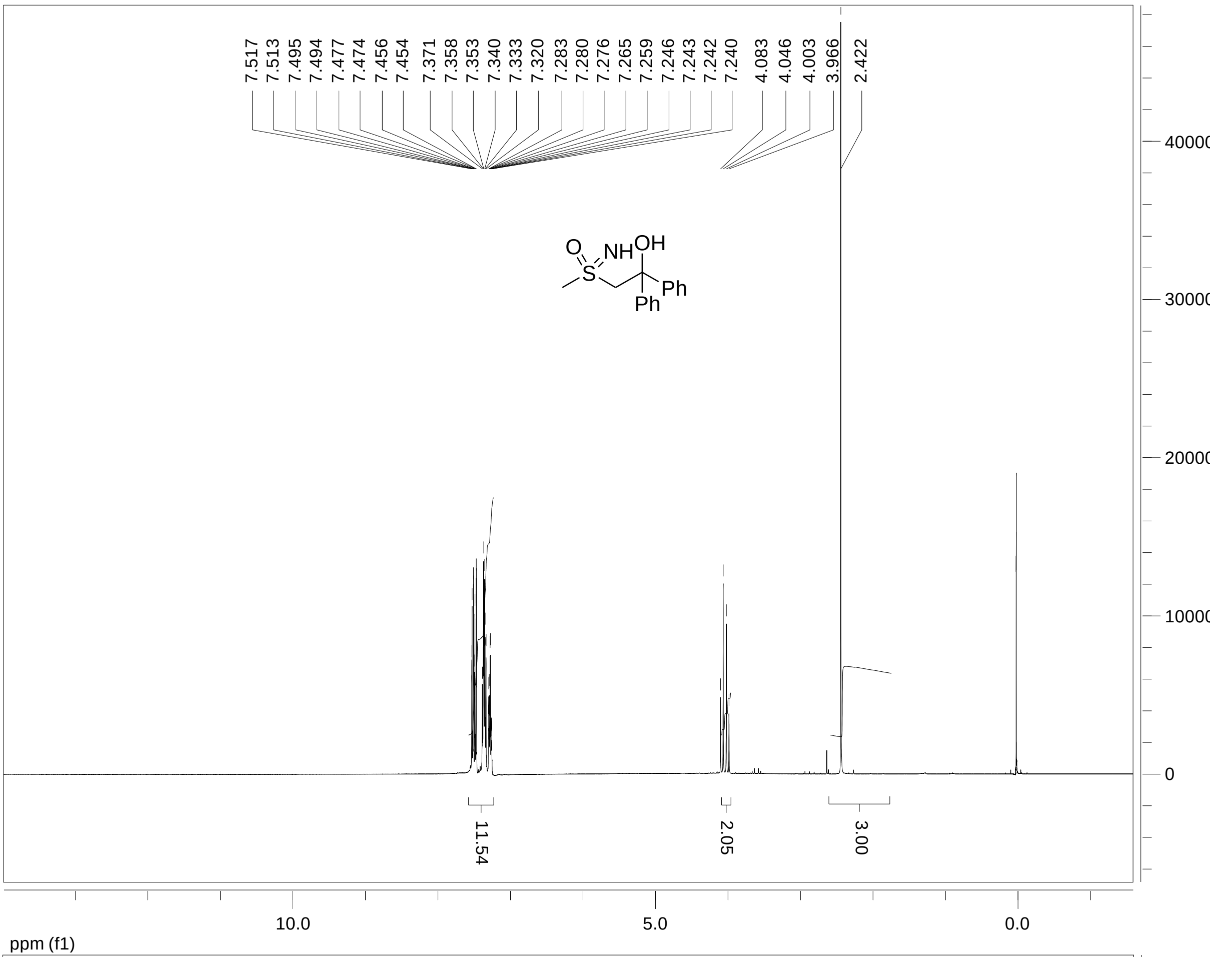
7.517 7.513 7.495 7.494 7.477 7.474 7.456 7.454 7.371 7.358 7.353 7.340 7.333 7.320 7.283 7.280 7.276 7.265 7.259 7.246 7.243 7.242 7.240 4.083 4.046 4.003 3.966 2.422
O
NH
OH
S
Ph
Ph
11.54
2.05
3.00
10.0
5.0
0.0
ppm (f1)

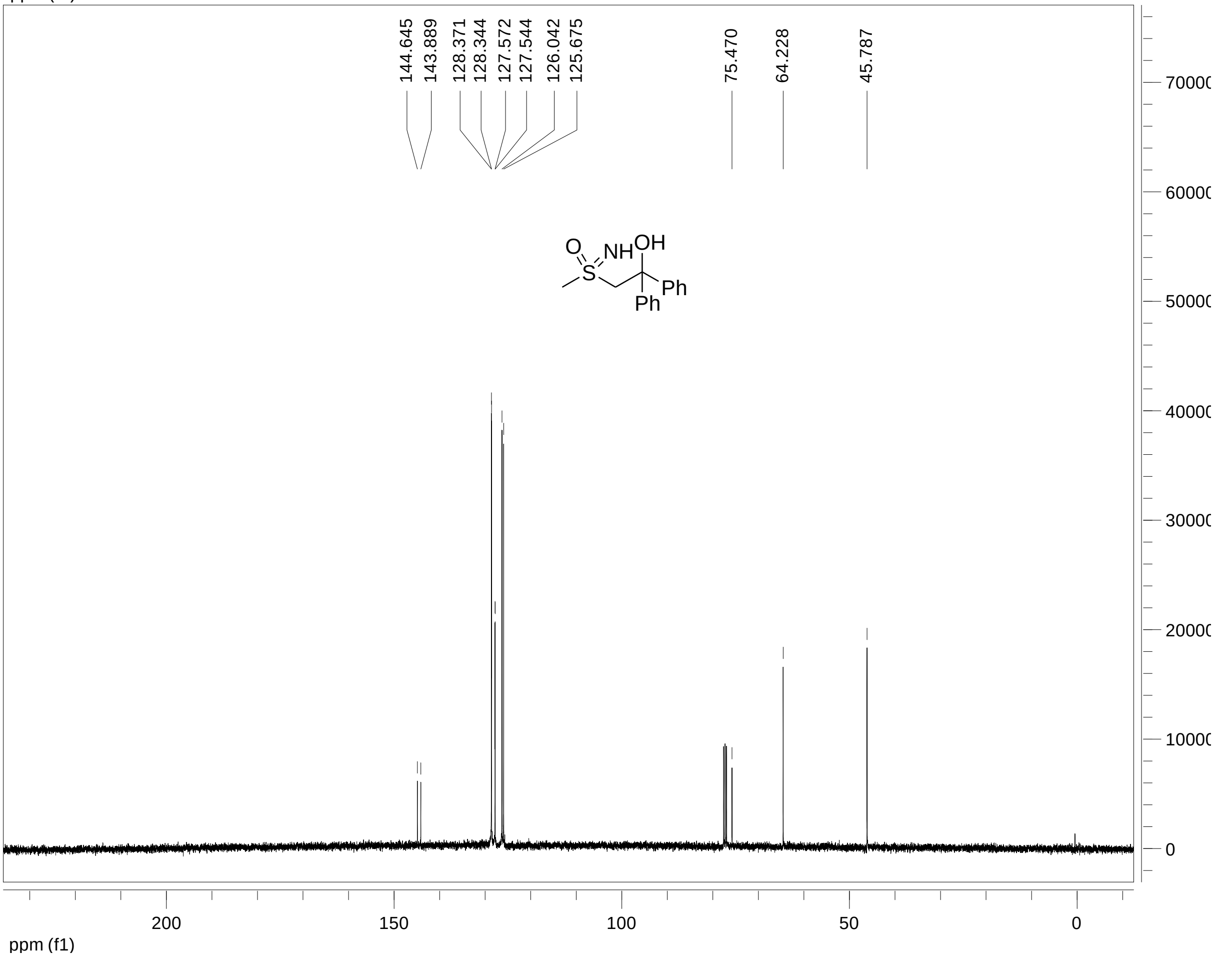
144.645 143.889 128.371 128.344 127.572 127.544 126.042 125.675 75.470 64.228 45.787
O
NH
OH
S
Ph
Ph
200
150
100
50
0
ppm (f1)

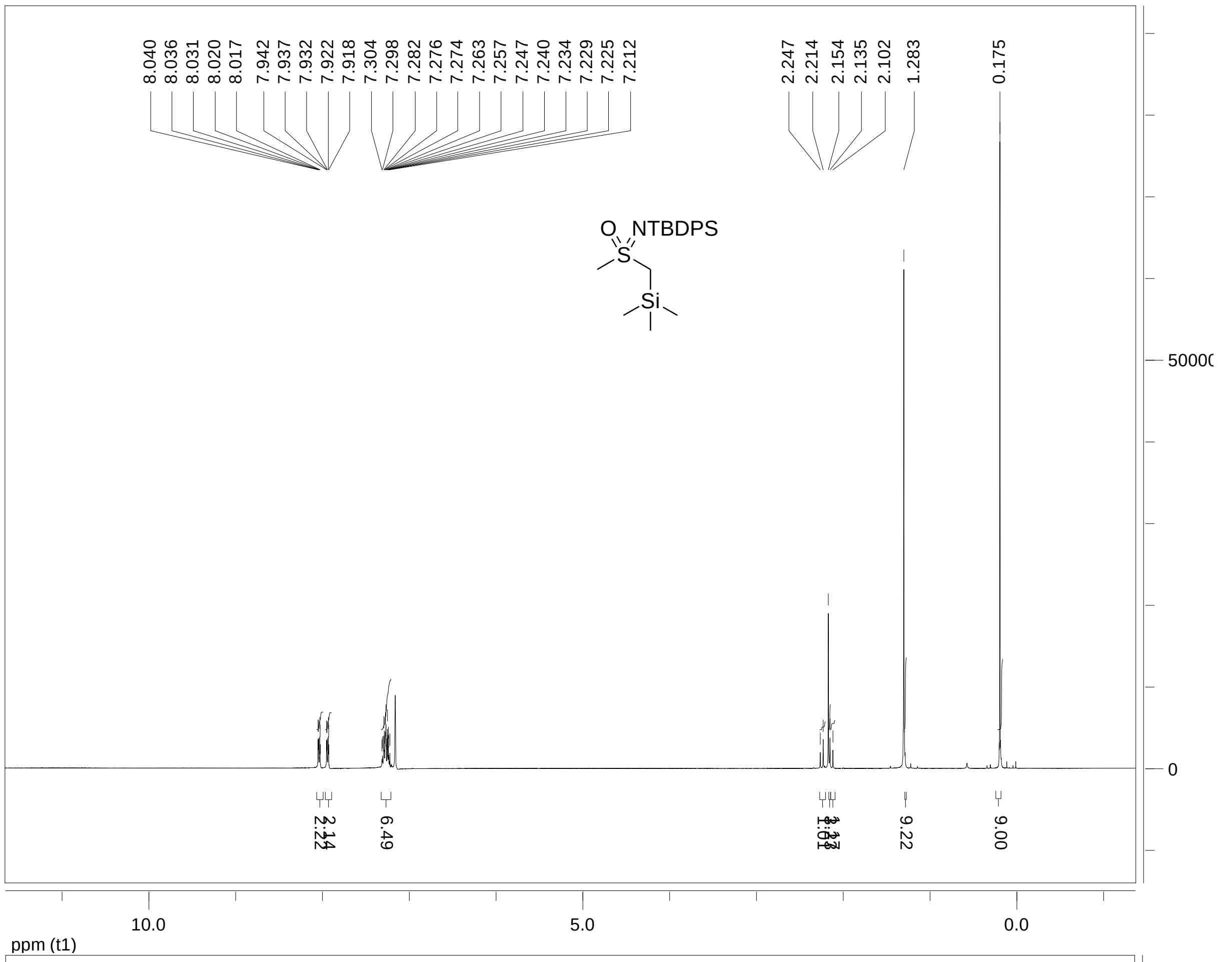
8.040
8.036
8.031
8.020
8.017
7.942
7.937
7.932
7.922
7.918
7.304
7.298
7.282
7.276
7.274
7.263
7.257
7.247
7.240
7.234
7.229
7.225
7.212
2.247
2.214
2.154
2.135
2.102
1.283
0.175
NTBDPS
O
S
Si
50000
0
2.22
2.14
6.49
9.22
9.00
10.0
5.0
0.0
ppm (t1)

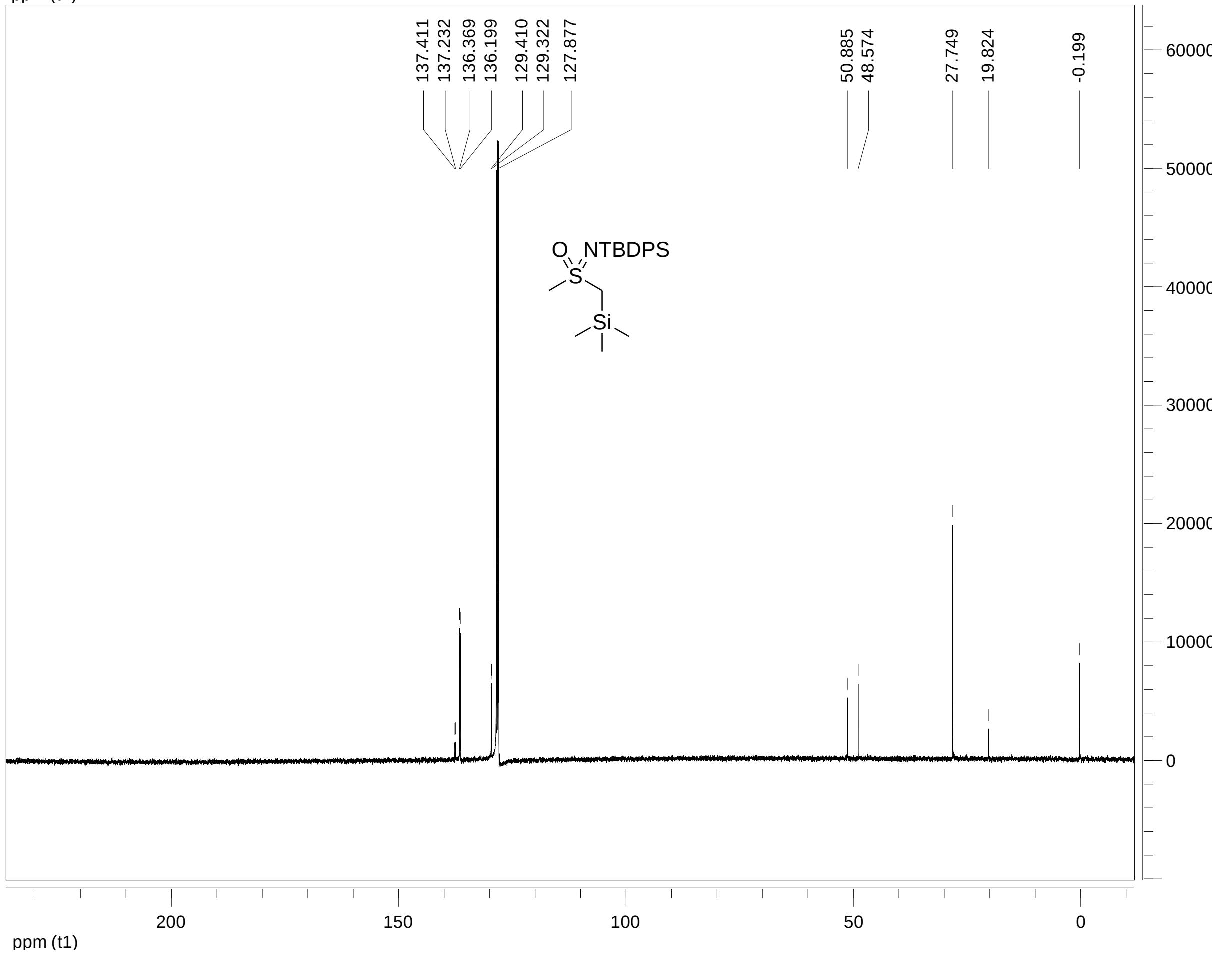
137.411
137.232
136.369
136.199
129.410
129.322
127.877
50.885
48.574
27.749
19.824
-0.199
O
NTBDPS
S
Si
60000
50000
40000
30000
20000
10000
0
200
150
100
50
0
ppm (t1)

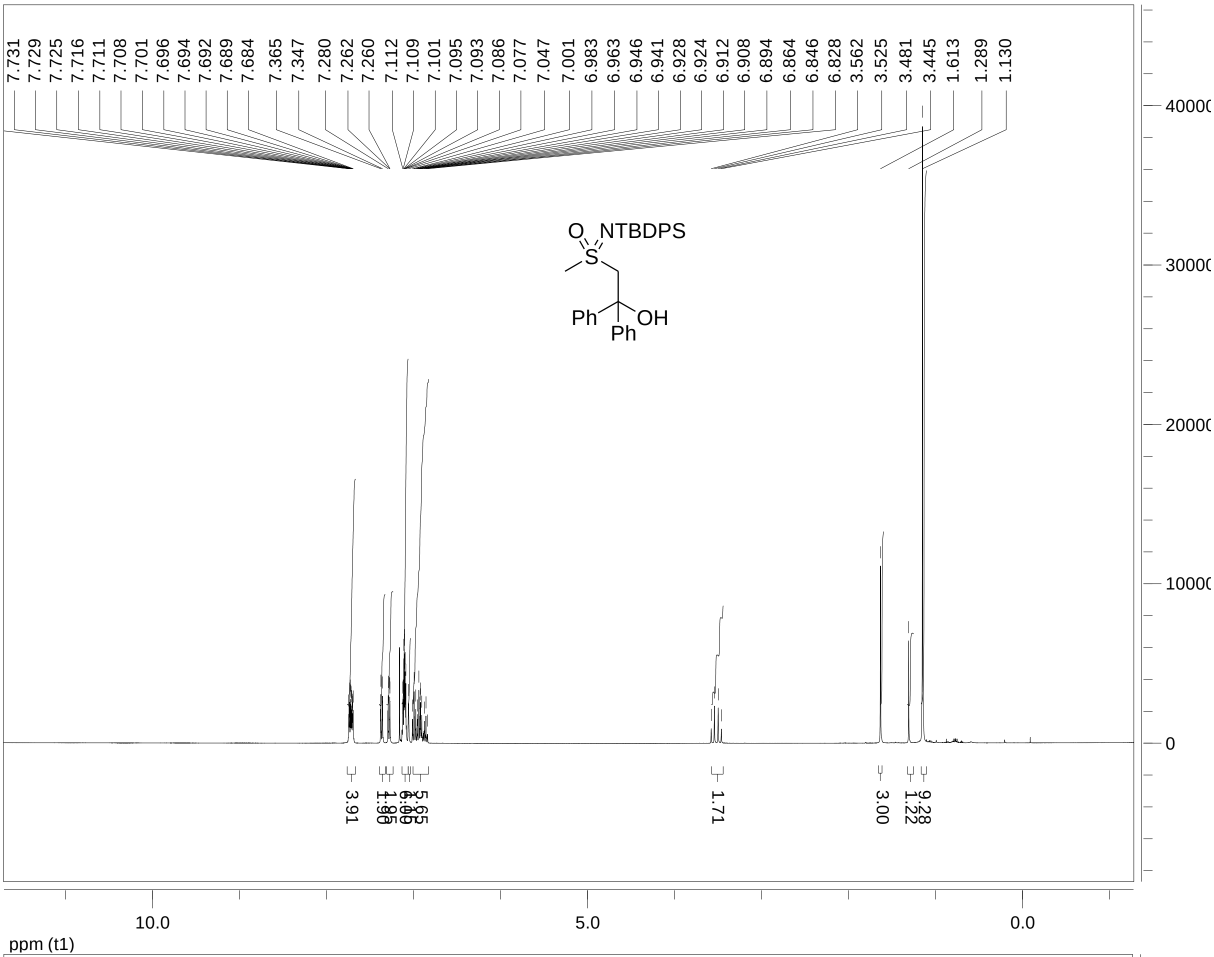

7.731 7.729 7.725 7.716 7.711 7.708 7.701 7.696 7.694 7.692 7.689 7.684 7.365 7.347 7.280 7.262 7.260 7.112 7.109 7.101 7.095 7.093 7.086 7.077 7.047 7.001 6.983 6.963 6.946 6.941 6.928 6.924 6.912 6.908 6.894 6.864 6.846 6.828 3.562 3.525 3.481 3.445 1.613 1.289 1.130
O NTBDPS
S
Ph OH
Ph
40000
30000
20000
10000
0
3.91
1.90
1.95
6.00
6.06
5.65
1.71
3.00
1.22
9.28
10.0
5.0
0.0
ppm (t1)

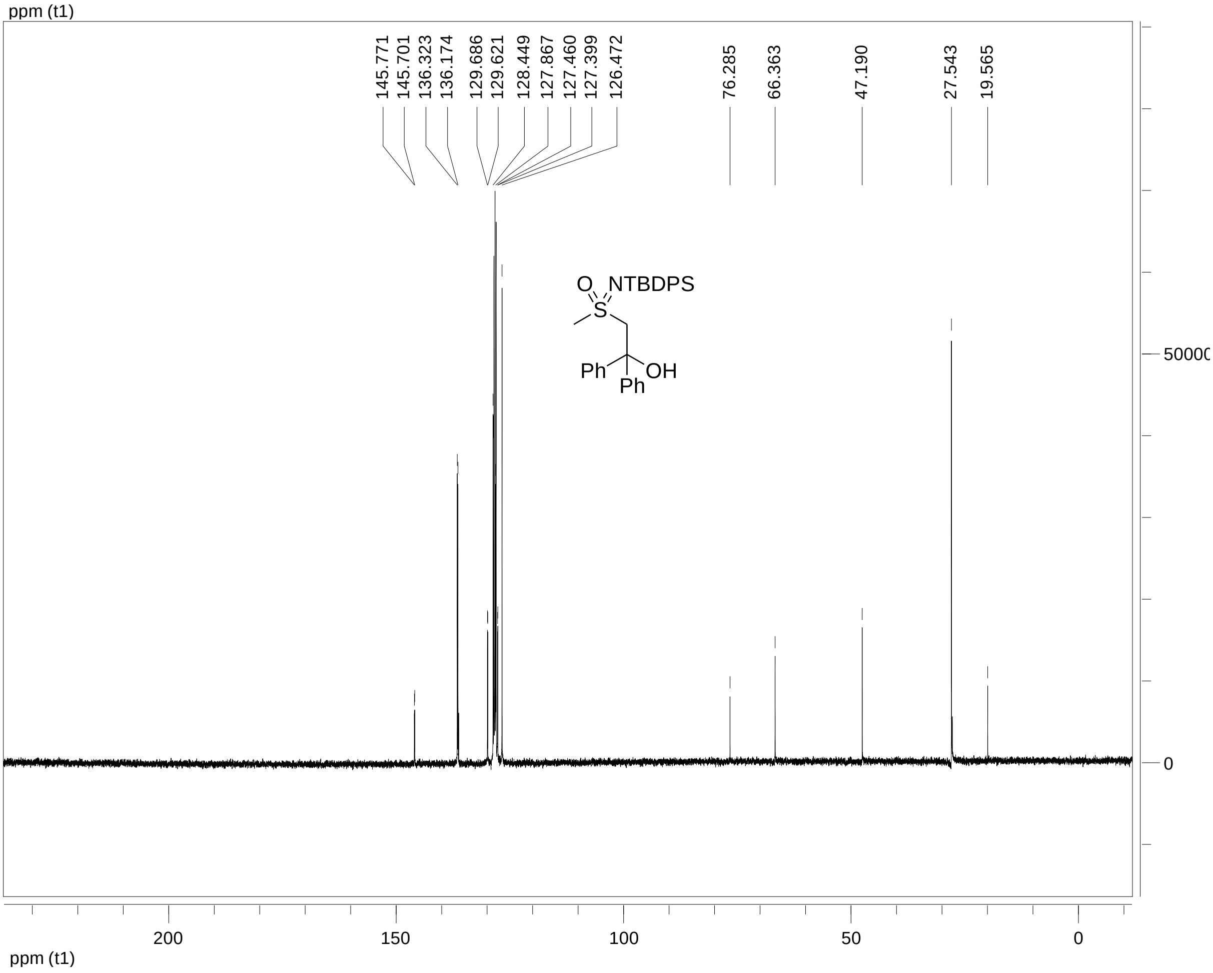

145.771 145.701 136.323 136.174 129.686 129.621 128.449 127.867 127.460 127.399 126.472
76.285
66.363
47.190
27.543
19.565
O NTBDPS
S
Ph OH
Ph
50000
0
200
150
100
50
0
ppm (t1)

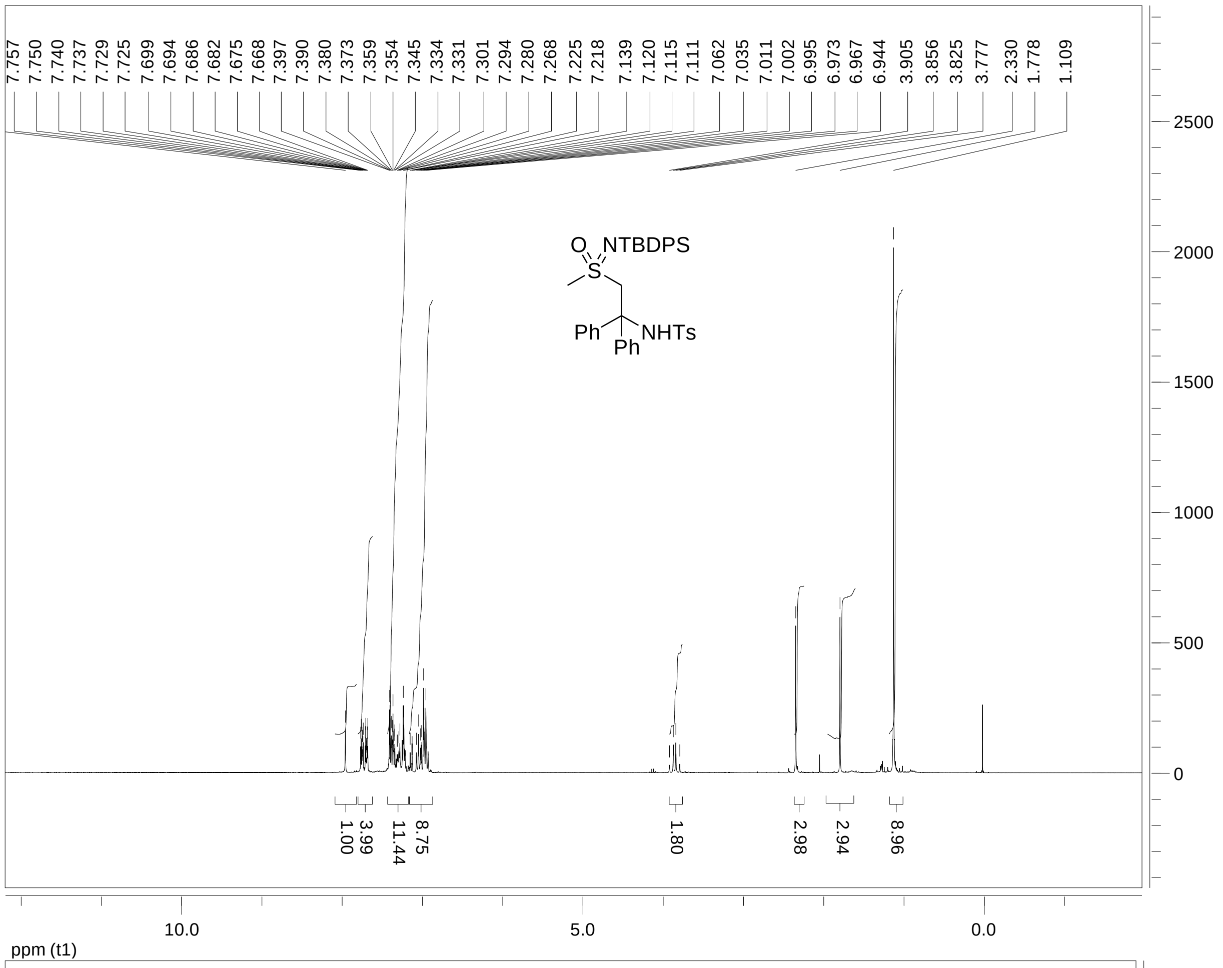

7.757 7.750 7.740 7.737 7.729 7.725 7.699 7.694 7.686 7.682 7.675 7.668 7.397 7.390 7.380 7.373 7.359 7.354 7.345 7.334 7.331 7.301 7.294 7.280 7.268 7.225 7.218 7.139 7.120 7.115 7.111 7.062 7.035 7.011 7.002 6.995 6.973 6.967 6.944 3.905 3.856 3.825 3.777 2.330 1.778 1.109
NTBDPS
NHTs
Ph
Ph
1.00 3.99 11.44 8.75 1.80 2.98 2.94 8.96
10.0
5.0
0.0
ppm (t1)

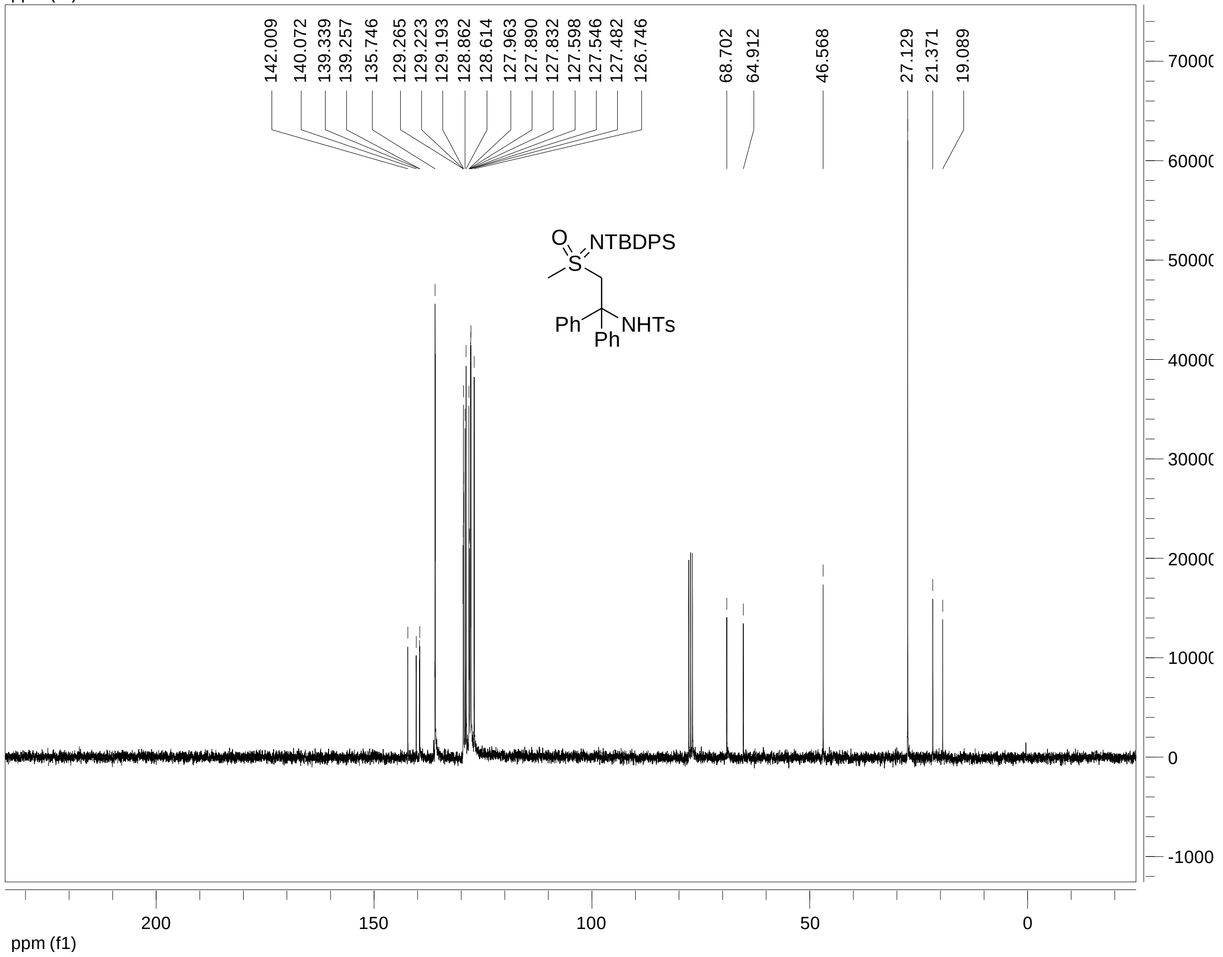

142.009 140.072 139.339 139.257 135.746 129.265 129.223 129.193 128.862 128.614 127.963 127.890 127.832 127.598 127.546 127.482 126.746 68.702 64.912 46.568 27.129 21.371 19.089
NTBDPS
NHTs
Ph
Ph
200
150
100
50
0
ppm (f1)

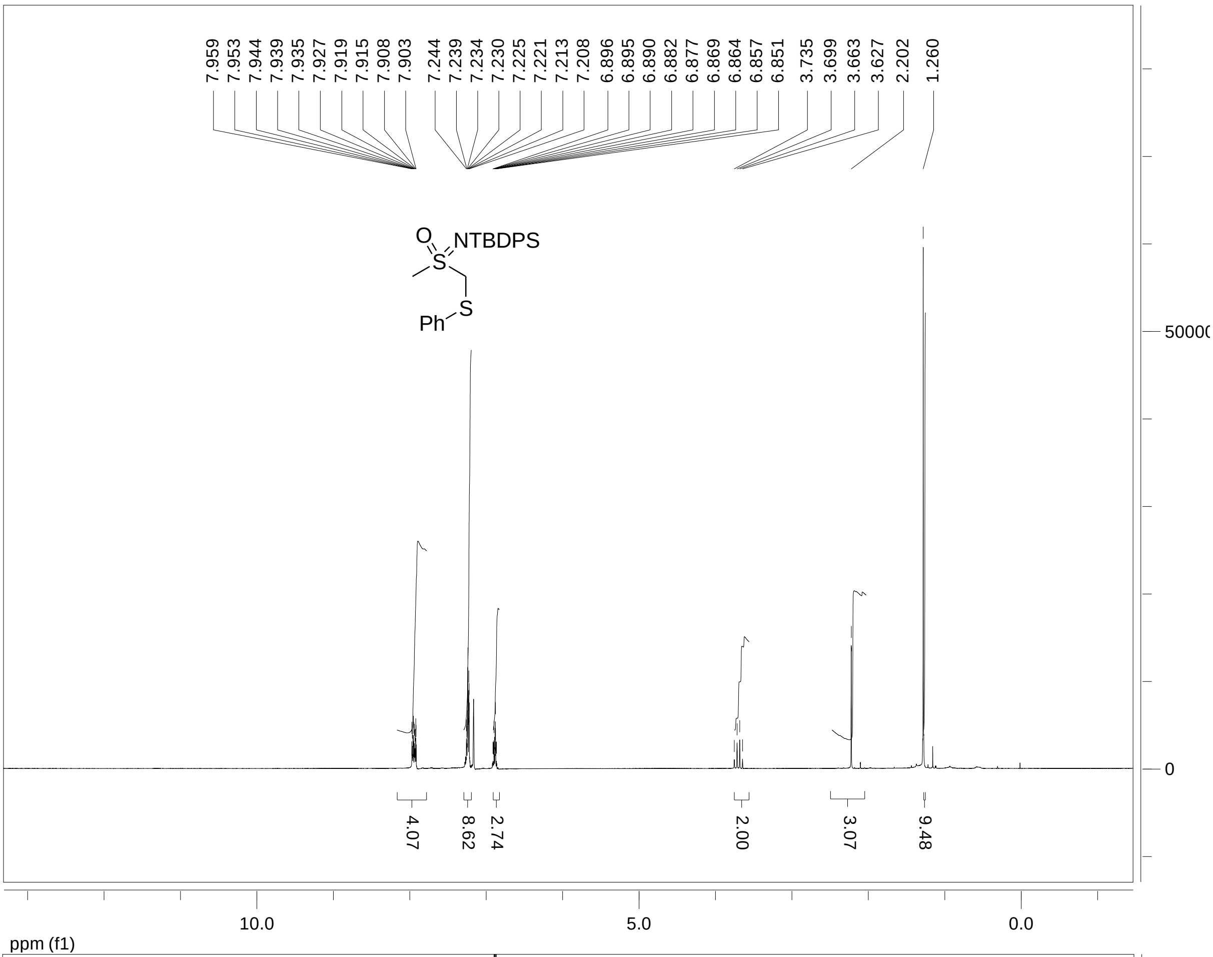
7.959
7.953
7.944
7.939
7.935
7.927
7.919
7.915
7.908
7.903
7.244
7.239
7.234
7.230
7.225
7.221
7.213
7.208
6.896
6.895
6.890
6.882
6.877
6.869
6.864
6.857
6.851
3.735
3.699
3.663
3.627
2.202
1.260
O
NTBDPS
S
S
Ph
4.07
8.62
2.74
2.00
3.07
9.48
50000
0
10.0
5.0
0.0
ppm (f1)

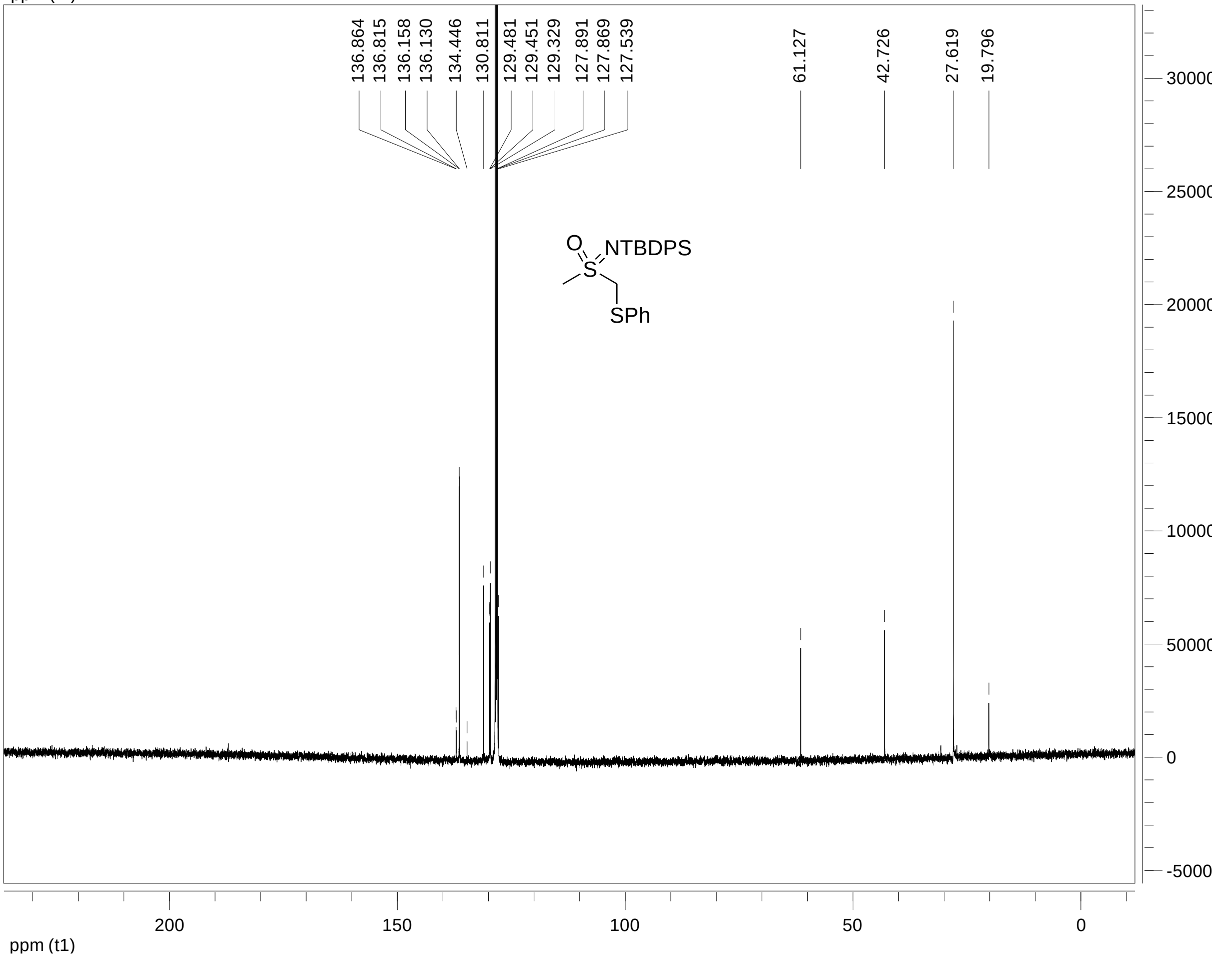
136.864
136.815
136.158
136.130
134.446
130.811
129.481
129.451
129.329
127.891
127.869
127.539
61.127
42.726
27.619
19.796
O
NTBDPS
S
SPh
30000
25000
20000
15000
10000
50000
0
-5000
200
150
100
50
0
ppm (t1)

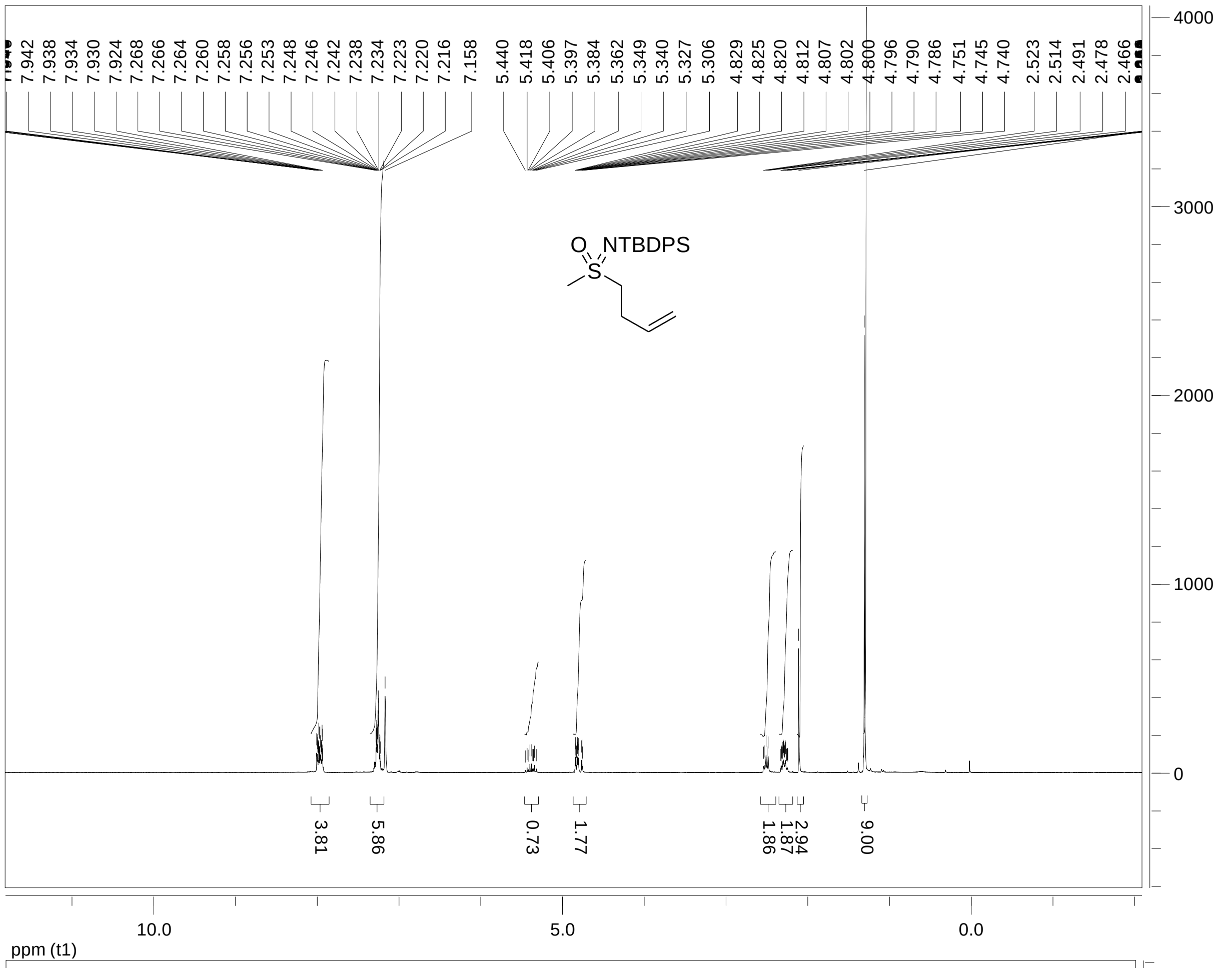
7.942
7.938
7.934
7.930
7.924
7.268
7.266
7.264
7.260
7.258
7.256
7.253
7.248
7.246
7.242
7.238
7.234
7.223
7.220
7.216
7.158
5.440
5.418
5.406
5.397
5.384
5.362
5.349
5.340
5.327
5.306
4.829
4.825
4.820
4.812
4.807
4.802
4.800
4.796
4.790
4.786
4.751
4.745
4.740
2.523
2.514
2.491
2.478
2.466
O
NTBDPS
S
3.81
5.86
0.73
1.77
1.86
1.87
2.94
9.00
4000
3000
2000
1000
0
10.0
5.0
0.0
ppm (t1)

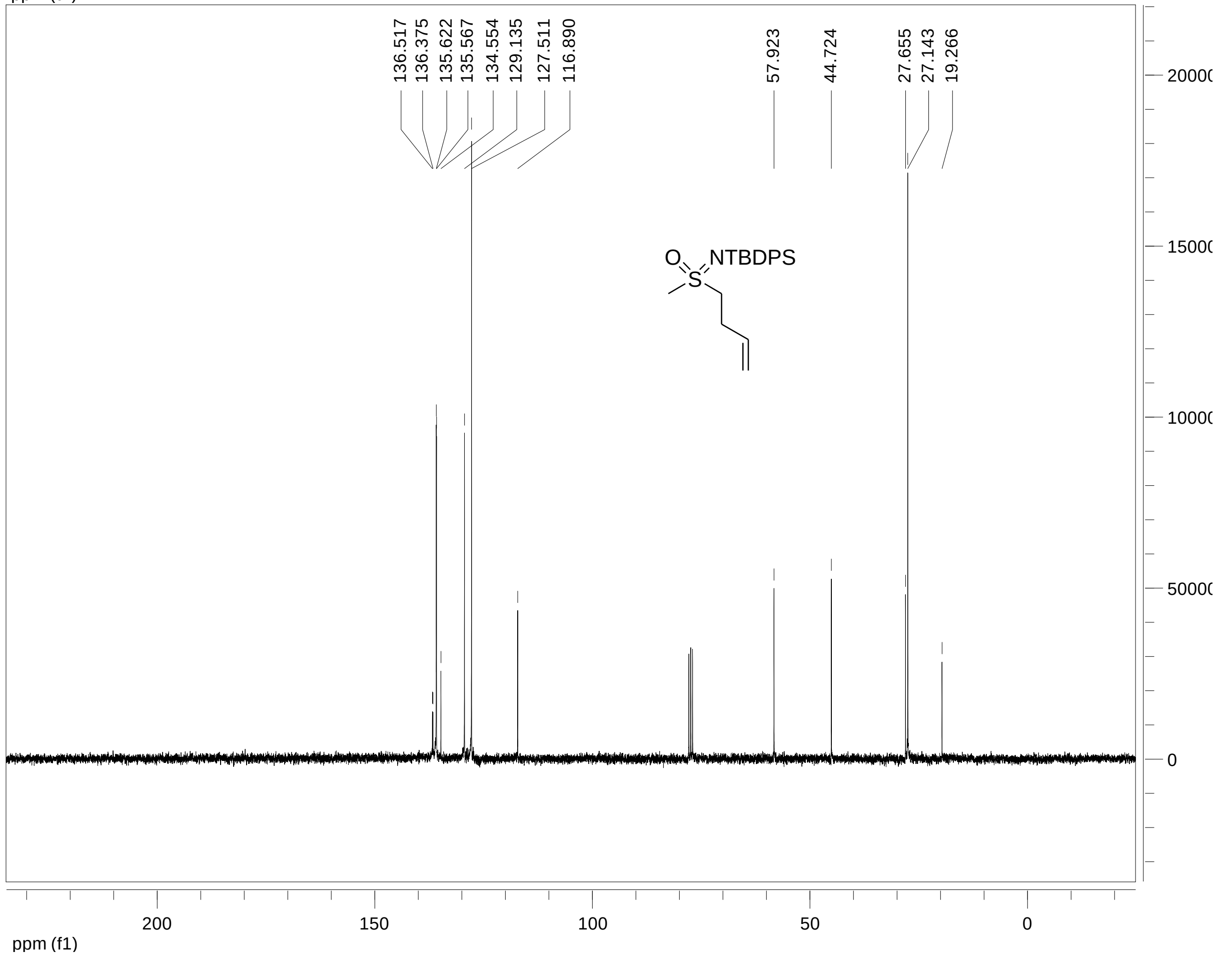
136.517
136.375
135.622
135.567
134.554
129.135
127.511
116.890
57.923
44.724
27.655
27.143
19.266
O
NTBDPS
S
200
150
100
50
0
ppm (f1)

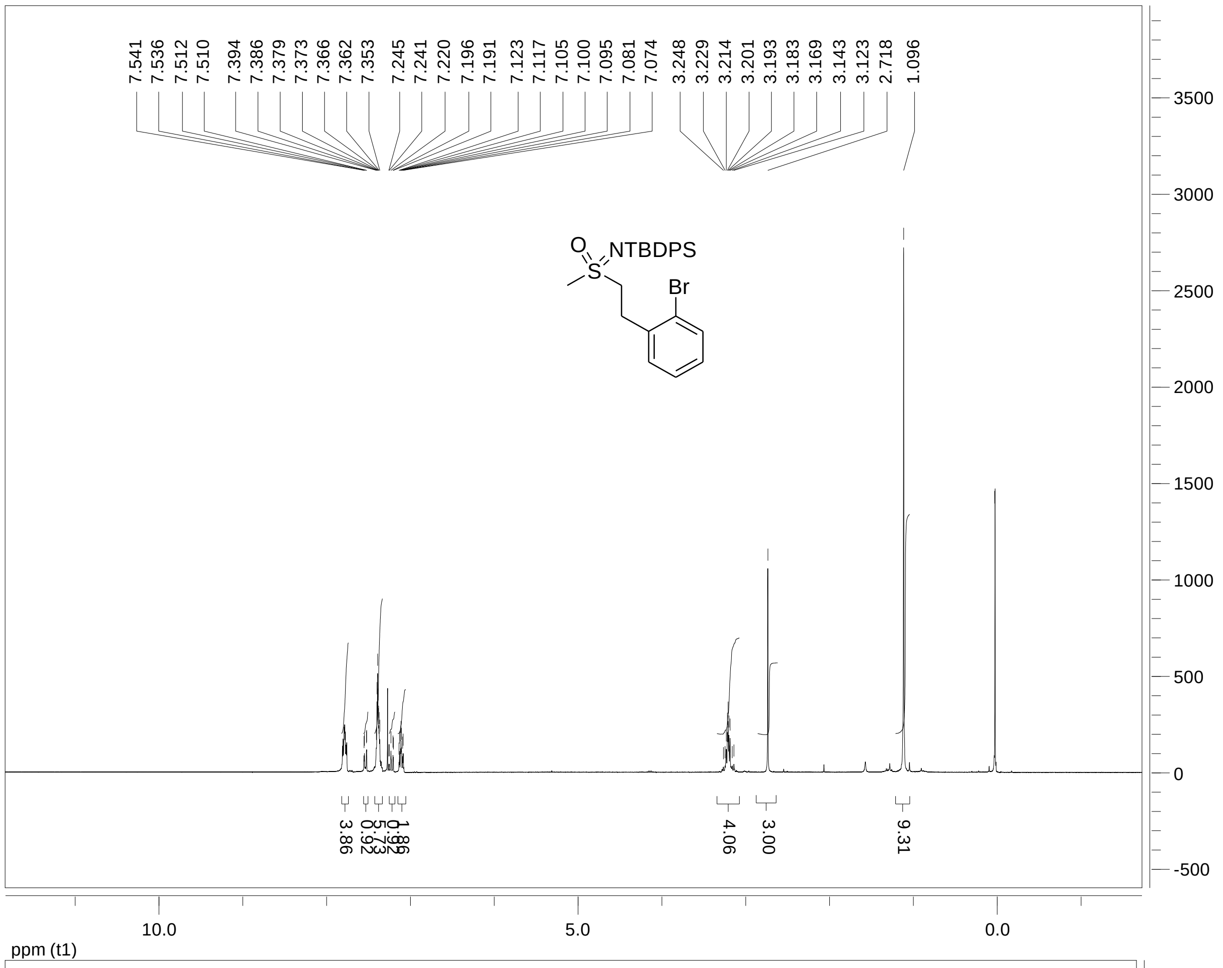
7.541
7.536
7.512
7.510
7.394
7.386
7.379
7.373
7.366
7.362
7.353
7.245
7.241
7.220
7.196
7.191
7.123
7.117
7.105
7.100
7.095
7.081
7.074
3.248
3.229
3.214
3.201
3.193
3.183
3.169
3.143
3.123
2.718
1.096
O
NTBDPS
S
Br
3.86
0.92
5.73
0.92
1.86
4.06
3.00
9.31
3500
3000
2500
2000
1500
1000
500
0
-500
10.0
5.0
0.0
ppm (t1)

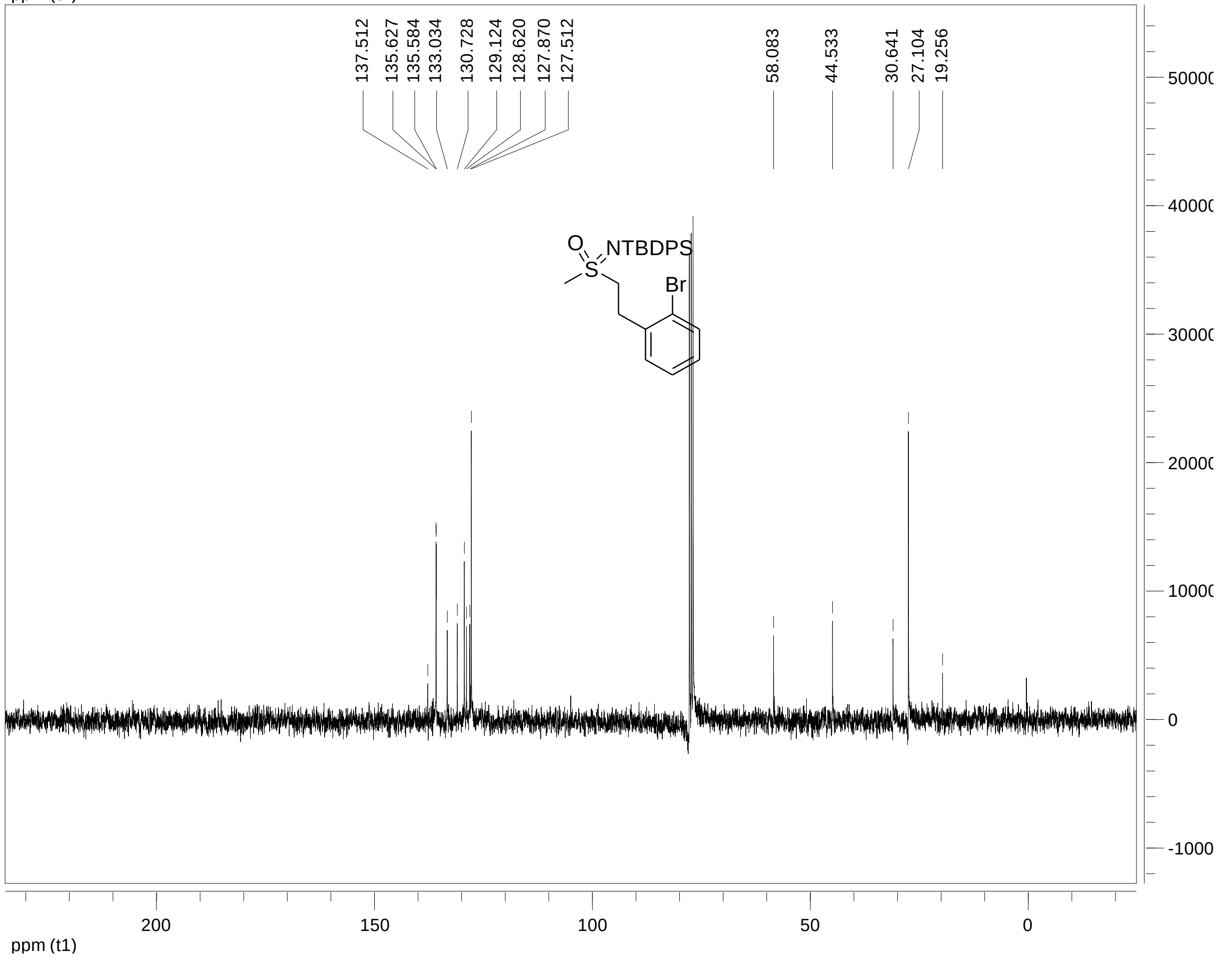
137.512
135.627
135.584
133.034
130.728
129.124
128.620
127.870
127.512
58.083
44.533
30.641
27.104
19.256
O
NTBDPS
S
Br
200
150
100
50
0
ppm (t1)

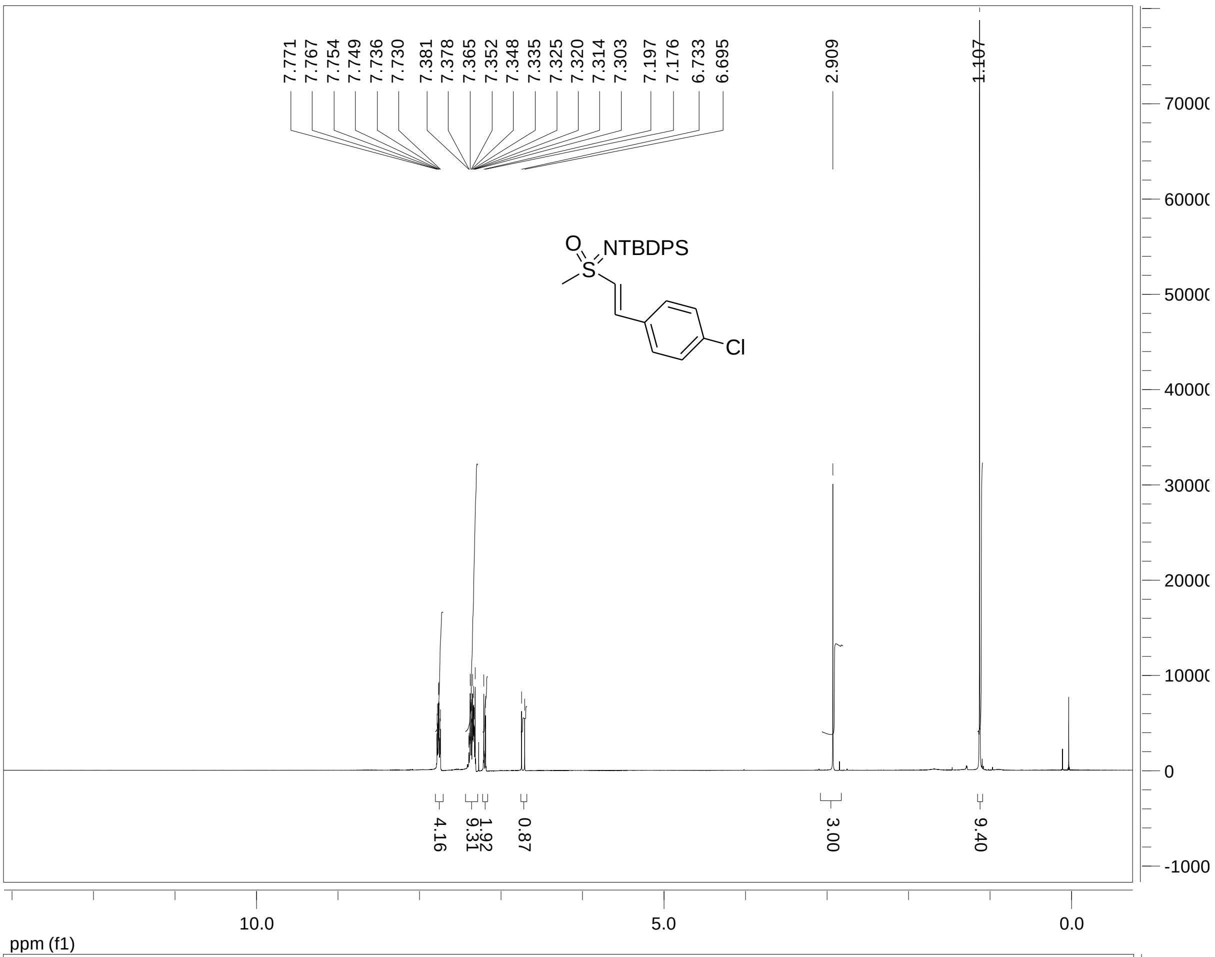

7.771 7.767 7.754 7.749 7.736 7.730 7.381 7.378 7.365 7.352 7.348 7.335 7.325 7.320 7.314 7.303 7.197 7.176 6.733 6.695
2.909
1.107
NTBDPS
O
S
Cl
4.16 9.31 1.92 0.87 3.00 9.40
10.0 5.0 0.0
ppm (f1)

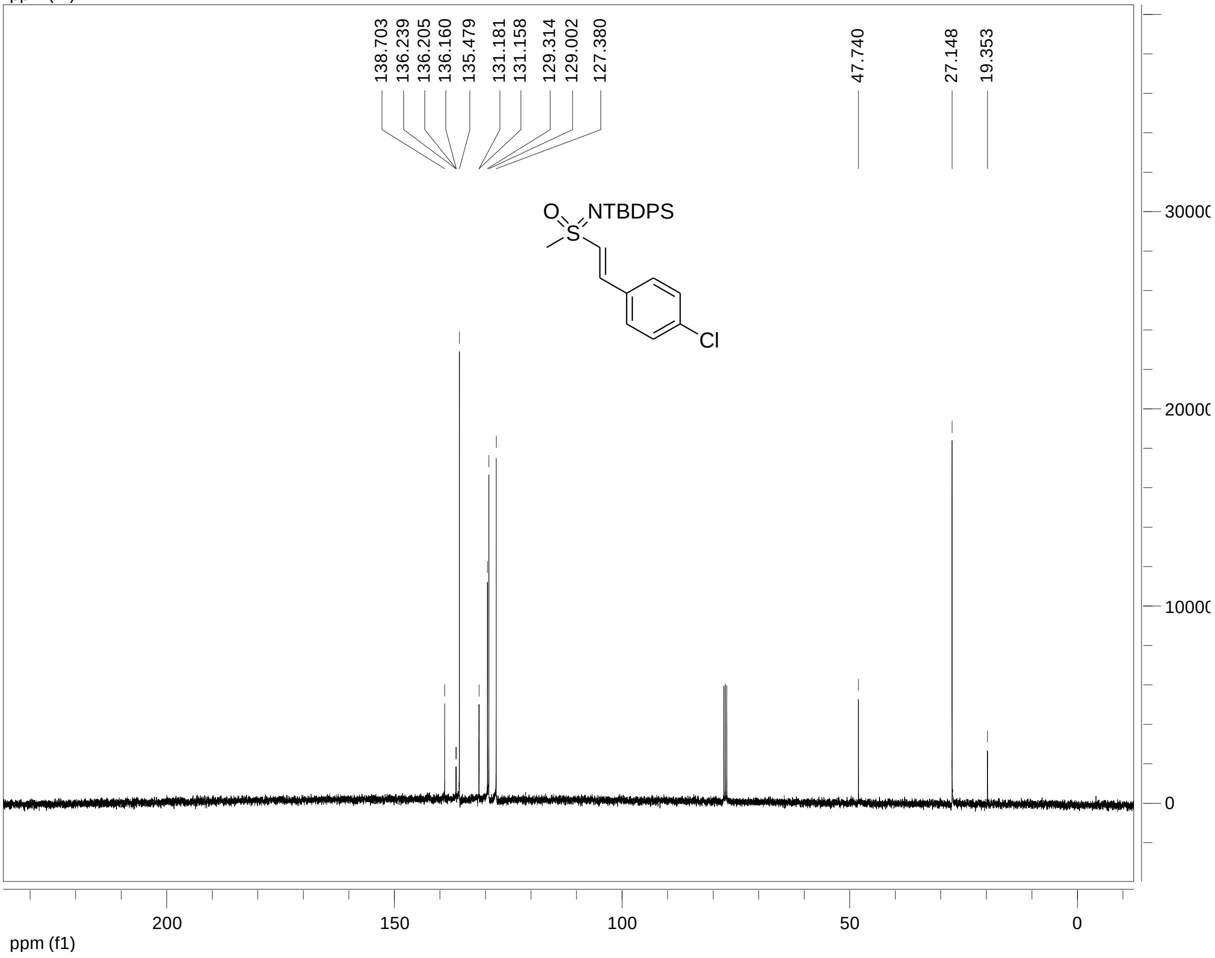

138.703 136.239 136.205 136.160 135.479 131.181 131.158 129.314 129.002 127.380
47.740
27.148 19.353
NTBDPS
O
S
Cl
200 150 100 50 0
ppm (f1)

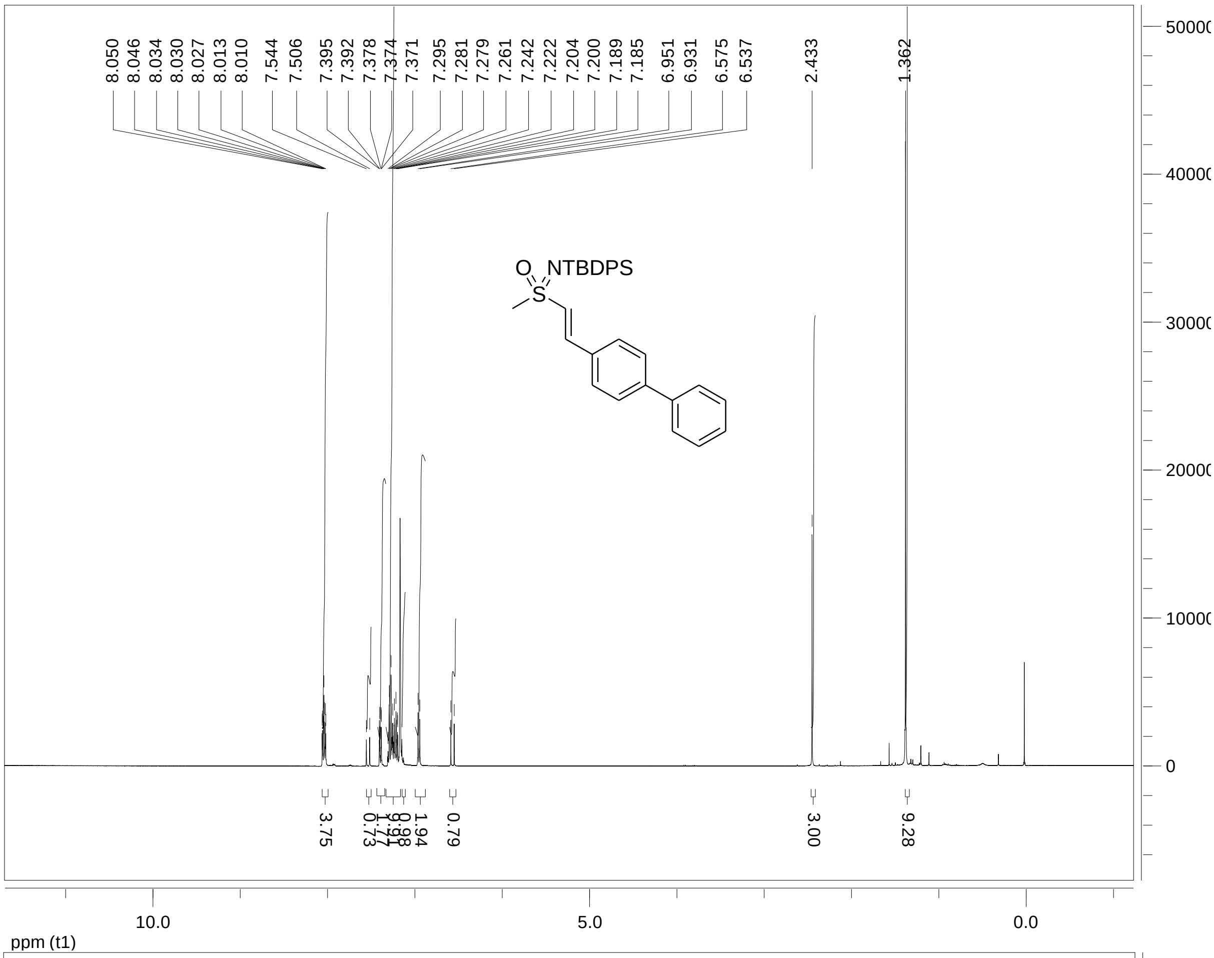
8.050 8.046 8.034 8.030 8.027 8.013 8.010 7.544 7.506 7.395 7.392 7.378 7.374 7.371 7.295 7.281 7.279 7.261 7.242 7.222 7.204 7.200 7.189 7.185 6.951 6.931 6.575 6.537
2.433
1.362
O
NTBDPS
S
3.75
0.73
1.77
1.91
0.98
1.94
0.79
3.00
9.28
10.0
5.0
0.0
ppm (t1)
50000
40000
30000
20000
10000
0

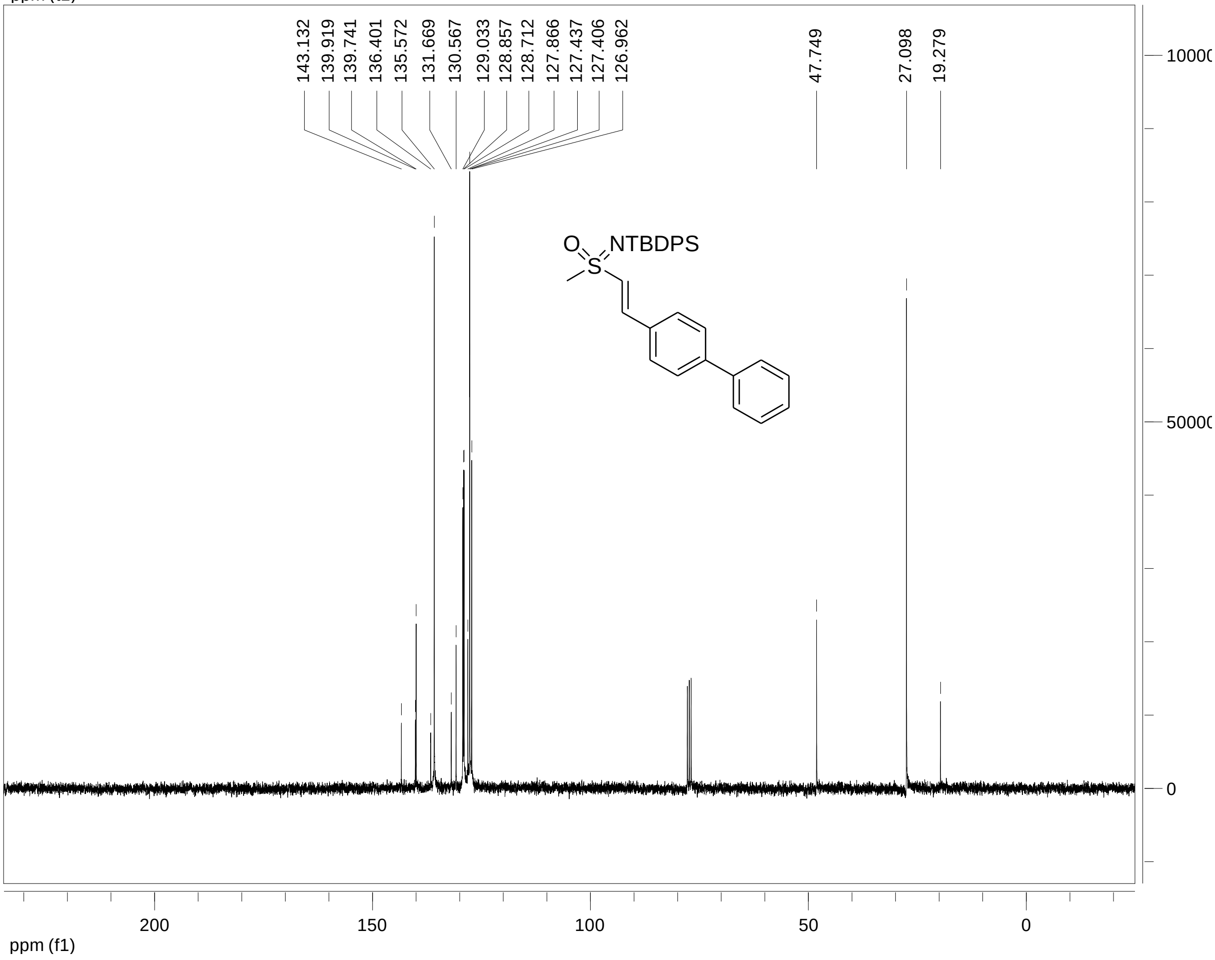
143.132 139.919 139.741 136.401 135.572 131.669 130.567 129.033 128.857 128.712 127.866 127.437 127.406 126.962
47.749
27.098
19.279
O
NTBDPS
S
200
150
100
50
0
ppm (f1)
10000
50000
0

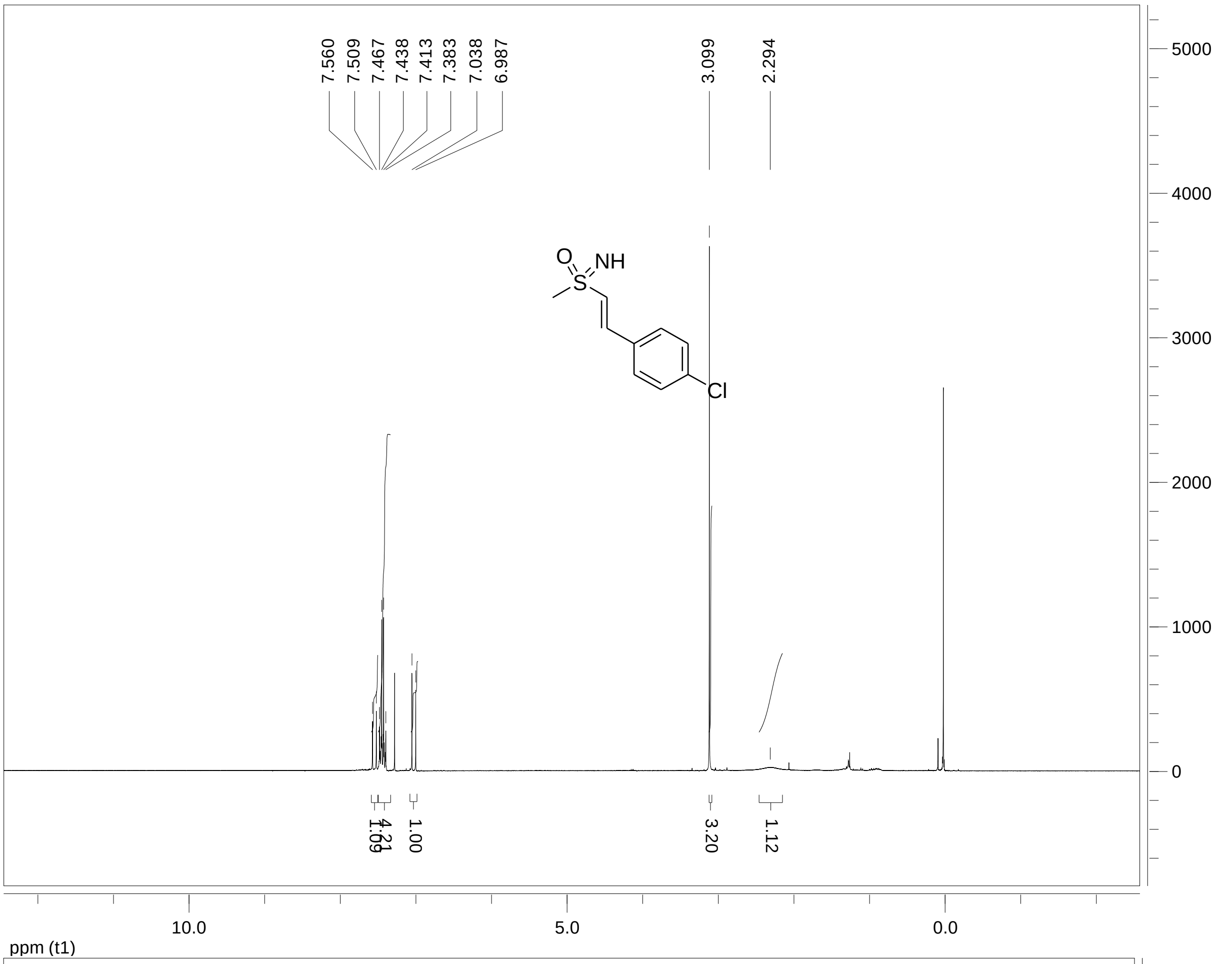

7.560
7.509
7.467
7.438
7.413
7.383
7.038
6.987
3.099
2.294
O
NH
S
Cl
1.09
4.21
1.00
3.20
1.12
5000
4000
3000
2000
1000
0
10.0
5.0
0.0
ppm (t1)

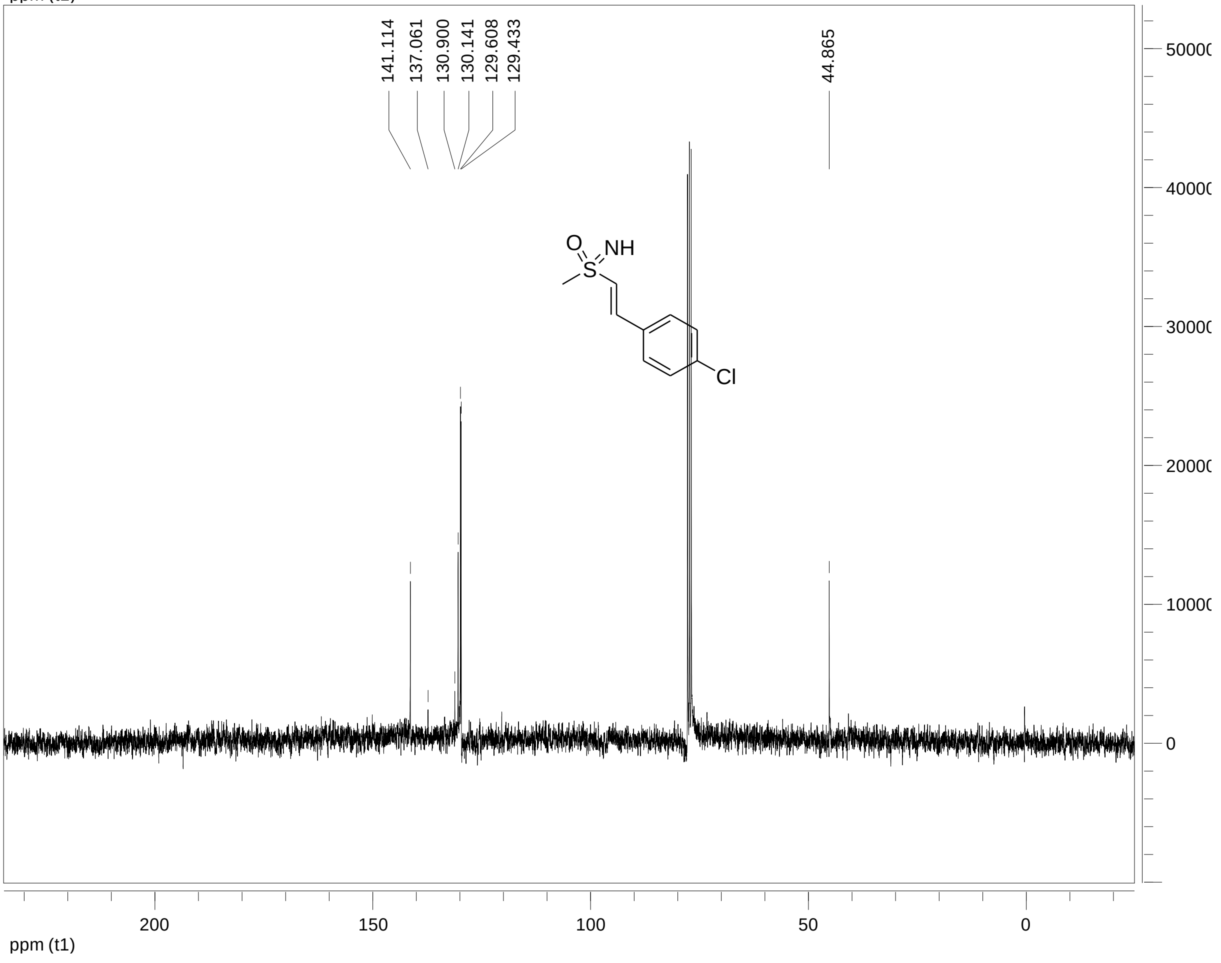

141.114
137.061
130.900
130.141
129.608
129.433
44.865
O
NH
S
Cl
200
150
100
50
0
ppm (t1)

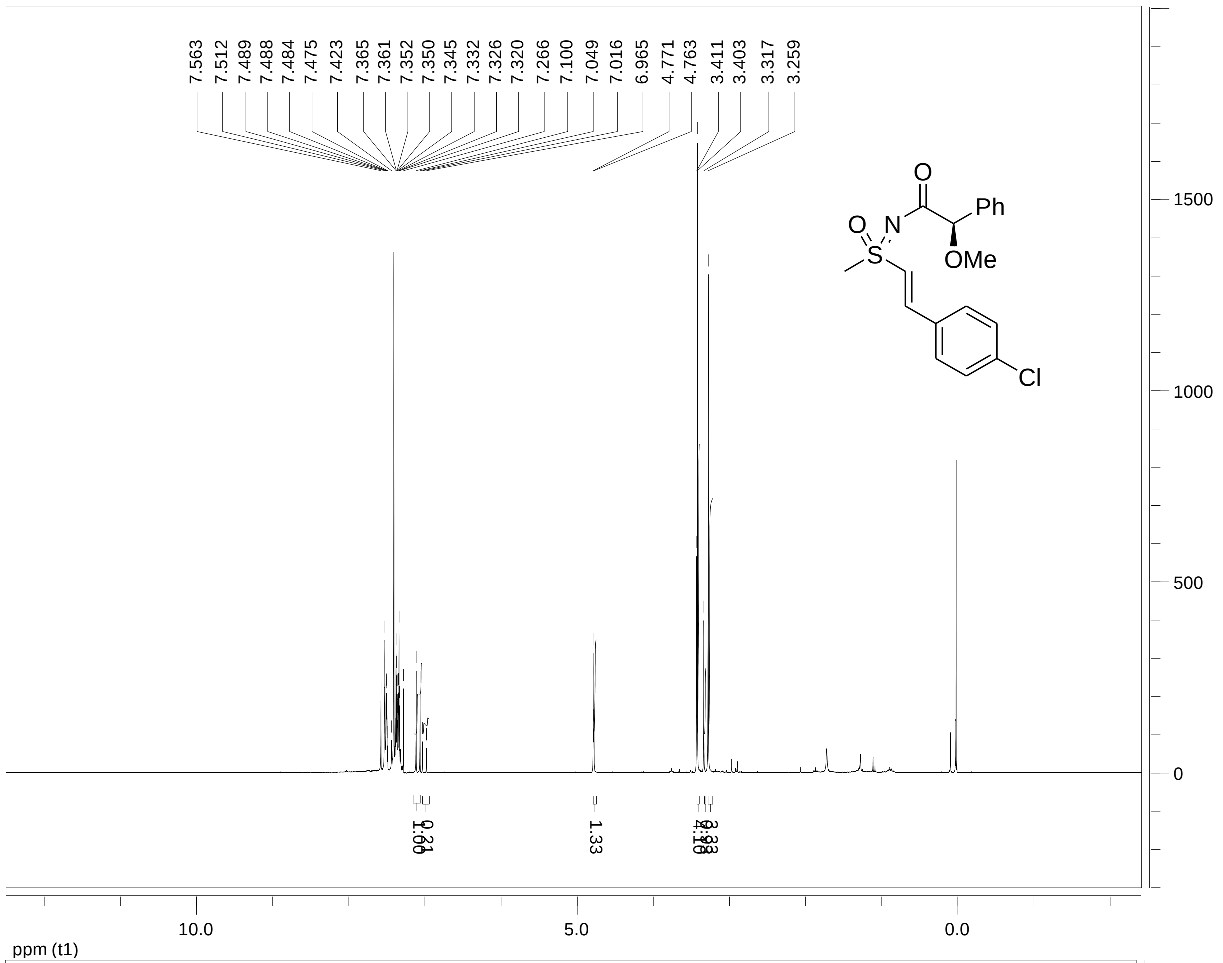
7.563
7.512
7.489
7.488
7.484
7.475
7.423
7.365
7.361
7.352
7.350
7.345
7.332
7.326
7.320
7.266
7.100
7.049
7.016
6.965
4.771
4.763
3.411
3.403
3.317
3.259
O
N
Ph
O
S
OMe
Cl
1500
1000
500
0
1.33
10.0
5.0
0.0
ppm (t1)

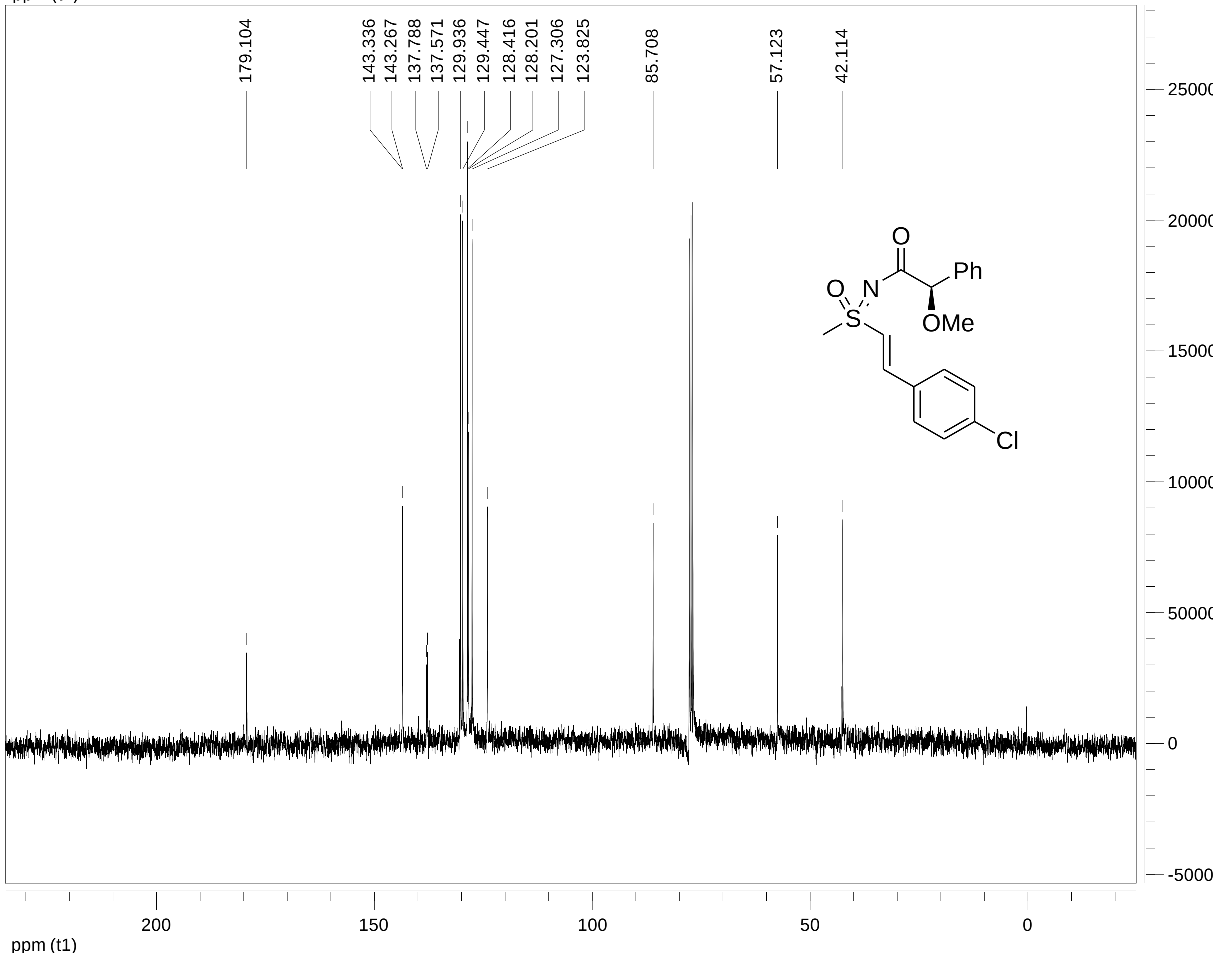
179.104
143.336
143.267
137.788
137.571
129.936
129.447
128.416
128.201
127.306
123.825
85.708
57.123
42.114
O
N
Ph
O
S
OMe
Cl
200
150
100
50
0
ppm (t1)
